# Tumor battlefield within inflamed, excluded or desert immune phenotypes: the mechanisms and strategies

**DOI:** 10.1186/s40164-024-00543-1

**Published:** 2024-08-06

**Authors:** Siwei Zheng, Wenwen Wang, Lesang Shen, Yao Yao, Wenjie Xia, Chao Ni

**Affiliations:** 1 Department of Breast Surgery, Second Affiliated Hospital, Zhejiang University School of Medicine, Hangzhou, 310000 China; 2 Key Laboratory of Tumor Microenvironment and Immune Therapy of Zhejiang Province, Second Affiliated Hospital, Zhejiang University School of Medicine, Hangzhou, 310000 China; 3 Department of Pathology, Second Affiliated Hospital, Zhejiang University School of Medicine, Hangzhou, 310000 China; 4 General Surgery, Cancer Center, Department of Breast Surgery, Zhejiang Provincial People’s Hospital (Affiliated People’s Hospital, Hangzhou Medical College), Hangzhou, 310000 China

**Keywords:** Tumor microenvironment, Tumor immune phenotype, Immunotherapy, Tumor metabolism

## Abstract

The tumor microenvironment demonstrates great immunophenotypic heterogeneity, which has been leveraged in traditional immune-hot/cold tumor categorization based on the abundance of intra-tumoral immune cells. By incorporating the spatial immune contexture, the tumor immunophenotype was further elaborated into immune-inflamed, immune-excluded, and immune-desert. However, the mechanisms underlying these different immune phenotypes are yet to be comprehensively elucidated. In this review, we discuss how tumor cells and the tumor microenvironment interact collectively to shape the immune landscape from the perspectives of tumor cells, immune cells, the extracellular matrix, and cancer metabolism, and we summarize potential therapeutic options according to distinct immunophenotypes for personalized precision medicine.

## Background

The past decades have witnessed encouraging practices in immunotherapy that have revolutionized the field of oncology by highlighting the host immune response as a viable target for cancer treatment. The most prospective approaches are immune checkpoint blockade (ICB) and adoptive cell therapy (ACT), as these strategies focus on key fighters in the anti-tumor battle [1]. ACT has shown remarkable achievements with six FDA-approved chimeric antigen receptor (CAR)-T cell therapies, particularly in hematologic malignancies in which a well-defined spatial structure is absent [2]. ICB primarily includes inhibitors of programmed cell death 1 (PD-1), programmed cell death ligand 1 (PD-L1) and cytotoxic T lymphocyte-associated antigen-4 (CTLA-4) [3]. As a monotherapy, ICB can yield beneficial and long-term therapeutic responses in patients with various types of cancer, with response rates ranging from 10 to 58% [4–6]. The mechanisms underlying such different likelihoods of response remain elusive and may be ascribed to the spatial heterogeneity of the preexisting tumor immune microenvironment (TIME) [7].

The TIME is composed of neoplastic cancer cells and non-cancer components, including multiple stromal and immune cells, vascular system, the extracellular matrix (ECM) compartments. Cancer cells actively orchestrate a tumor-supportive microenvironment, whereas the evolving TIME selects certain tumor subclones with survival advantages [8, 9]. This complex interplay is partly reflected in the immune landscape, which is considered the focal point of the TIME [10–14]. Traditionally, it is believed that the number of tumor-infiltrating lymphocytes (TILs) serves as a predictor for immunotherapy susceptibility and prognosis; therefore, tumors are dichotomized into immune-hot (abundant infiltration of CD8^+^ T cells) and immune-cold (limited infiltration of CD8^+^ T cells) phenotypes [15–17]. The Immunoscore is a worldwide accepted and standardized scoring system for colorectal cancer (CRC) that quantifies the density of CD3^+^ and CD8^+^ T cells within the tumor center and invasive margin. By introducing immune parameters, the Immunoscore has been validated to outperform other prognostic indicators, including pathologic T and N stages, lymphovascular invasion, tumor differentiation, and microsatellite instability (MSI) status [18, 19]. Nonetheless, the GeparNeuvo trial (NCT02685059), which was stratified by the quantity of stromal tumor-infiltrating lymphocytes (sTILs) before neoadjuvant chemotherapy, showed that sTIL status is not statistically significant in predicting invasive disease-free survival and pathological complete response, emphasizing the need for comprehensive knowledge of distinct spatial patterns of the TIME [20].

A decade before, Chen and Mellman proposed a novel trichotomic classification of tumor immunophenotypes. Based primarily on the spatial and quantity distribution of immune cells (CD8^+^ T cells in particular) within tumor nest or stromal compartments, the TIME can be morphologically defined into “immune-inflamed”, “immune-excluded” and “immune-desert” with distinctive traits [13, 21, 22]. The Impassion130 (NCT02425891) trial revealed a declining tendency of PD-L1 expression in tumor cells following the aforementioned order, indicating a significant difference in immunotherapy susceptibility across the three immunophenotypes [23]. The immune-excluded phenotype, newly identified from the traditional “hot/cold” classification, has been unveiled to be associated with ICB resistance [24]. One plausible explanation for this phenomenon is that activated CD8^+^ T cells are excluded from the tumor parenchyma and, therefore, cannot effectively kill malignant cells owing to their limited ability to penetrate the tumor core. However, a comprehensive knowledge of the factors contributing to different immunophenotypes remains to be elucidated. In this review, we summarize the interrelationships between tumor cells and the TIME that underlie distinctive immunophenotypes. The ultimate goal was to identify the biologicals vulnerabilities of cancer and provide a rationale for precise anti-tumor treatments.

## Immune features of the Inflamed, excluded and Desert TIME (Fig. 1)

**Fig. 1:**
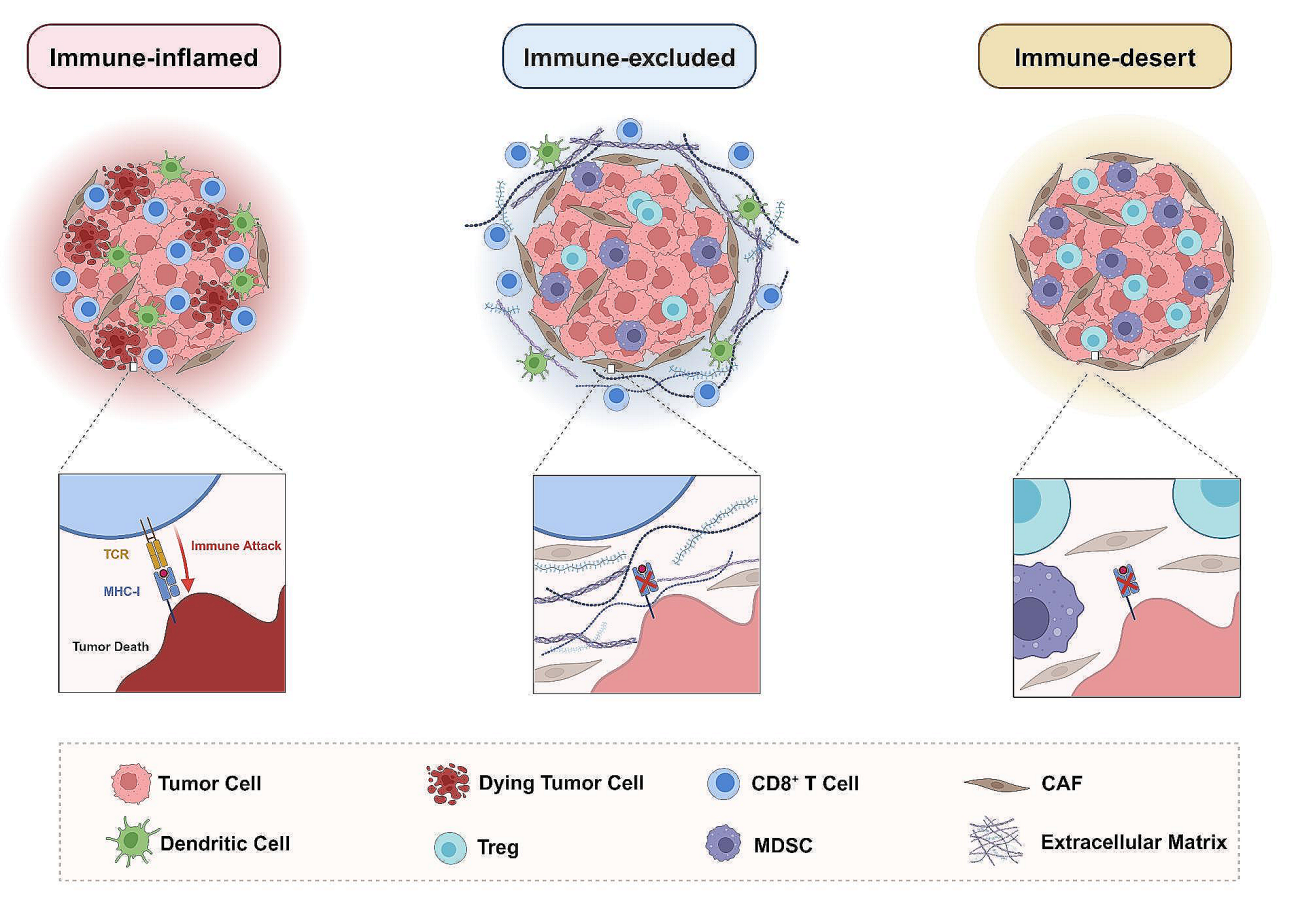
The Schematic Diagram of Immune-inflamed, Immune-excluded, and Immune-desert Tumors. The immune-inflamed tumors are characterized by an abundant infiltration of CD8^+^ T cells within the tumor parenchyma, which involves active tumoricidal immune attacks. While for the immune-excluded tumors, CD8^+^ cells are generally trapped in the peritumoral stroma, failing to directly eliminate tumor cells. As for the immune-desert tumors, CD8^+^ T cells are barely present either in the parenchymal or stromal sites, instead, immune suppressive cells (e.g. Tregs, MDSCs) may abound in the TIME

Conventionally, immune-inflamed tumors (so-called “hot” or “immune-infiltrated” tumors) are characterized by a profusion of TILs both in the tumor nests and stroma. Patients with inflamed tumors, which account for up to 50% of all human tumors, generally portend favorable response towards chemotherapy and ICB [21, 25–27]. The immune-inflamed phenotype is associated with elevated genomic instability and antigenicity [21], along with an accumulation of proinflammatory cytokines and an increased interferon response; however, whether the inflammatory TIME is the cause or consequence of immune cell influx remains an open question [25].

Immune-desert (also referred to as “cold” or “ignored”) tumors, as the name suggests, indicate a paucity or absence of T cells either in the tumor core or periphery, though myeloid cells may be present instead [21, 25, 28, 29]. This immunophenotype features defective antigen presentation machinery (APM) and exhibits a reduced interferon (IFN) response, as well as an expansion of immunosuppressive cells [21, 30–32]. As reported, chemotherapy or ICB treatment remains dismal towards immune-desert tumors.

Finally, immune-excluded tumors show a distinctive T-cell immune context [25]. CD8^+^ T cells are located in the vicinity of the tumor parenchyma but are incapable of penetrating and having direct dialogue with tumor cells. Instead, immune cells circumferentially “stuck” in the peritumoral fibroblast- and collagen-rich stroma [21, 29, 33, 34]. This implies ineffective T cell activation, proliferation, and trafficking [25]. Given its failure to mount an efficient immune response, the efficacy towards ICB is generally inferior to that of immune-inflamed tumors, although some indicate an even worse prognosis than the desert immunophenotype, which is of significant clinical relevance [35–37]. In an analysis of the ICOL7 trial ovarian cancer cohort, patients with immune-excluded phenotypes had shorter progression-free survival than those with either inflamed or desert phenotypes [38]. The biological mechanisms underlying the immune-excluded phenotype remain inconclusive [28, 38] (Fig. 1).

**Fig. 2:**
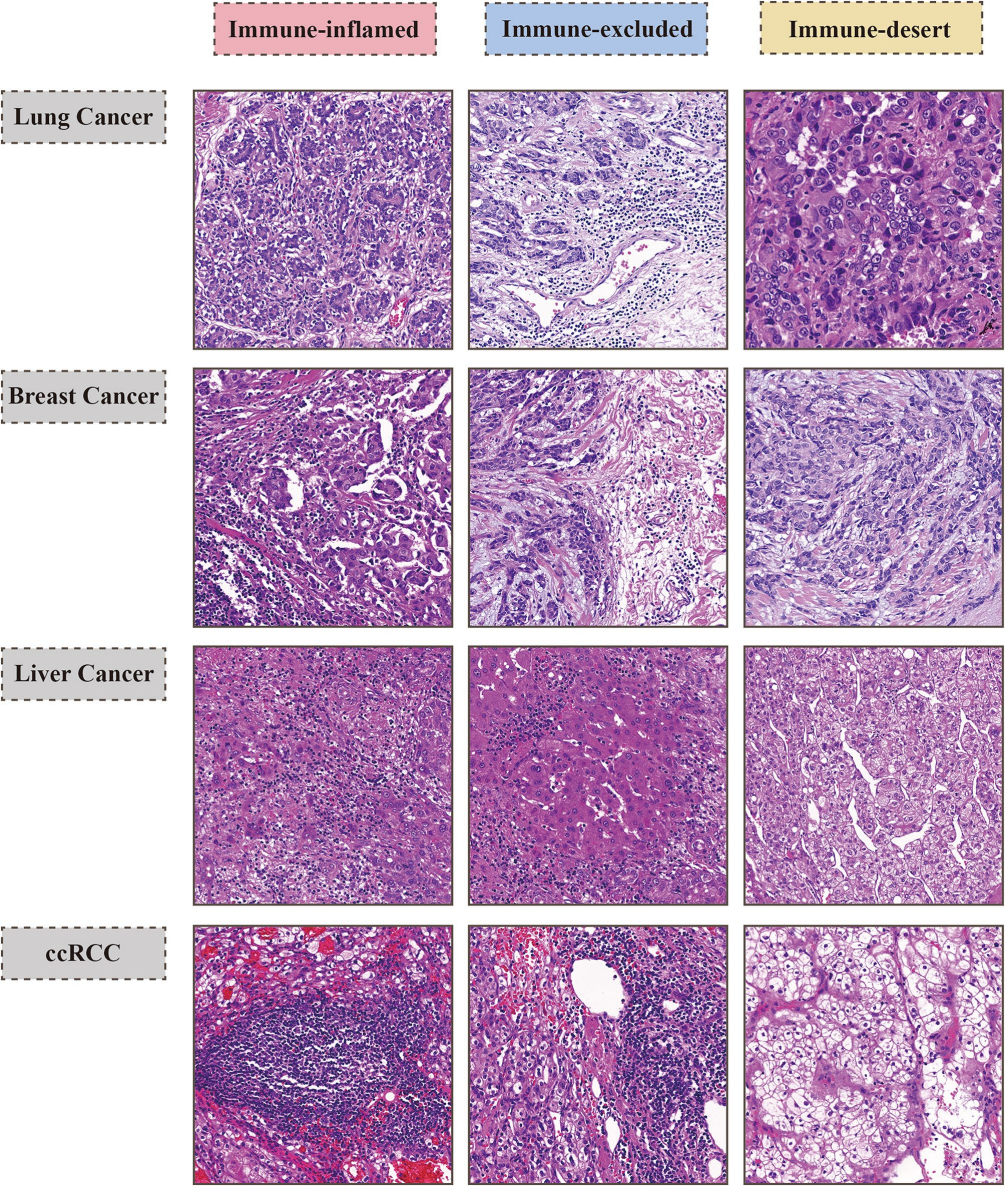
Representative HE Images of Corresponding Tumor Immune Phenotypes from Lung Cancer, Breast Cancer, Liver Cancer, and Clear Cell Renal Cell Carcinoma

The phenotypic classification of the TIME is complicated by inter- and intra-tumoral heterogeneity, which appeals for a clear-cut consensus to drive further applications **(**Fig. 2**)** [39, 40]. Currently, Immunohistochemistry (IHC) is the most commonly used technique for evaluating immune infiltration patterns as it allows for the quantification of immune cells in terms of their type, density, and distribution [37]. However, an important question is whether human tissues, which are intrinsically three-dimensional (3D), are examined as limited two-dimensional cross-sections that may potentially misrepresent the entire tumor landscape due to sampling bias [41]. Technical constraints have hindered the holistic characterization of TIME, as a larger tissue volume mitigates sampling bias and accounts for tissue heterogeneity [42]. Recently, a 3D pathology deep learning platform, TriPath, has demonstrated superior prognostic performance over traditional two-dimensional slice-based approaches, indicating its potential clinical applications in defining tumor immunophenotypes [41]. Furthermore, immune monitoring challenges persist, as immune parameters are dynamically altered during tumor progression [43]. One viable diagnostic procedure, immunopositron-emission tomography imaging, holds great promise for the non-invasive tracking of intra-tumoral CD8^+^ T cells [44]. In addition, other authors have constructed gene signature-based categorizations using transcriptome analysis [45, 46]. For instance, genes enrichment of IFN-γ pathway are usually recognized as “inflamed” tumors and signatures of stromal biology are indicative for immune-excluded ones, while absence of both is categorized as immune-desert [33, 47–49]. Several techniques based on the deconvolution of bulk gene expression data have been developed to predict the level of intra-tumoral immune infiltrates, including CIBERSORT (which estimates the proportional distribution of immune subsets within the overall leukocyte population) [46, 50]; xCell (which evaluates the abundance of immune cells within the TIME) [51]; TIMER (which calculates enrichment scores by analyzing the proportions of immune and stromal cell types) [52] and integrated immunogenomics methods that can be employed to identify immune subtypes of cancer with a CIBERSORT-based approach [48]. Nevertheless, these immunophenotype-predicting techniques have inevitable limitations in terms of inconsistency during the RNA extraction procedure, the impossibility of univocally assigning transcripts to specific cell subsets, and discrepancies in immunophenotypes between circulating blood and the TIME across cancer types. However, the high cost of novel single-cell based approaches [53] and in situ barcode sequencing [54] hinders their large-scale diagnostic applications.

Notably, these immunophenotype classification methods are not simply one-size-fits-all methods; some tumors possess attributes that span multiple categories [55]. Desbois et al. have highlighted that the quantity and landscape of immune cells within the TIME are continuous [38]. In other words, the immunophenotypes of specific tumor types differ among individuals and should be comprehensively evaluated on a case-by-case basis **(**Table 1**)**. CRCs are primarily composed of the immune-excluded subtype with up to 70–75% frequencies, while only 10% of cases exhibit an inflamed TIME. Conversely, non-small cell lung cancers (NSCLC) show approximately 40% excluded and 30–35% inflamed phenotype [13]. Breast cancer (BC) is histologically purported as a “cold” tumor type while in-depth research has uncovered its strong immunophenotypic heterogeneity among subtypes [56, 57]. Basal-like subtype represents the largest fraction of the “inflamed” BC, followed by HER-2 and Luminal-B tumors. Triple negative breast cancer (TNBC) was typically considered as the most immunogenic subtype, however, approximately 28% and 26% of TNBC cases exhibit an immune-desert and immune-excluded pattern respectively, clearly contradicting the notion that TNBC is “inflamed” [58]. Moreover, the metastasized TNBC exhibits a divergent pattern from primary lesions, consisting of higher proportions of the excluded (41%) and desert (37%) phenotypes [58]. For now though, due to the great heterogeneity across solid tumors, as well as a lack of reliable biomarkers, the definitions of inflamed, excluded and desert tumor types are far from consistent. An increasing number of studies have explored the mechanisms underlying various TIME subtypes during tumorigenesis. Here, we comprehensively review the pertinent literatures that is expected to improve treatment strategies based on tumor immunophenotypes for tailored-comers in the future.

**Table 1:** Proportions of immune-inflamed, immune-excluded, and immune desert subtypes from different tumors

Cancer Type	Immune-inflamed	Immune-excluded	Immune-desert	Reference
TNBC	46%	26%	28%	10.1038/s41467-021-25962-0
NSCLC	35%	40%	25%	10.1016/j.immuni.2019.12.011
Pancreatic Cancer	44%	46%	10%	10.3390/ijms25010142
CRC	10%	75%	15%	10.1016/j.immuni.2019.12.011
Ovarian Cancer	27%	45%	28%	10.3390/cancers14174246
mUC	26%	47%	27%	10.1038/nature25501
mTNBC	22%	41%	37%	10.1038/s41467-021-25962-0
HCC	31%	24%	45%	10.7150/jca.54408
ccRCC	22%	19%	59%	10.1038/s41379-021-00864-0

*Abbreviations*: TNBC: Triple Negative Breast Cancer; NSCLC: Non-small Cell Lung Cancer; CRC: Colorectal Cancer; mUC: metastatic Urothelial Cancer; mTNBC: metastatic Triple Negative Breast Cancer; HCC: Hepatocellular Carcinoma; ccRCC: clear cell Renal Cell Carcinoma

## The mechanisms of anti-tumor immune anergy in different TIME

Tumor antigen recognition initiates the cancer-immunity cycle, triggering cascades that involve antigen presentation, immune activation, cytotoxic effector cell trafficking, infiltration into the tumor nest, and the recognition and destruction of cancer cells [25, 59, 60]. Lynch syndrome (LS) is a hereditary CRC syndrome caused by germline mutations in the DNA repairing machinery. The consequent excessive mutational load and neoantigen levels contribute to the enrichment of TILs within the TIME, resulting in a striking immune inflammation [61]. Despite the adaptive immune system, physical barriers may pose hurdles to the direct elimination of cancer. Fibrous tissues and stromal components encase the tumor tightly, whereas internal tumor endothelial cells may exhibit a dysfunctional morphology and phenotype, rendering them unresponsive to inflammatory signals. In this context, CD8^+^ T-cell trafficking and infiltration into tumor nests are obstructed. Overall, the status of the TIME could be modulated by any step in the cancer immunity cycle, which results in a specific tumor immunophenotype and implies available therapeutic targets.

### Genomic instability and TILs heterogeneity

Genomic instability (GI) drives tumor evolution, which is conceptually measured by tumor mutational burden (TMB) and MSI [62]. The U.S. food and drug association has proposed high tumor mutational burden (TMB-H) at a cutoff value of ≥ 10 mut/Mb, which serves as a prognostic predictor of survival rates and ICB responses for solid tumor patients [63]. This suggests a positive correlation between genomic alterations and higher immunogenicity, potentiating cancer-responsive TILs [64–68]. Immune-inflamed tumors typically present with a high TMB status or deficiencies in DNA repair mechanisms, such as mismatch repair deficiency, and subsequent MSI. These characteristics increase the neoantigen load and attract TILs to the tumor parenchyma [69–71]. Studies on various hypermutated malignancies, including melanoma and lung, bladder, and urothelial cancers, have provided compelling evidence of an inflamed immune microenvironment [33, 64, 72]. However, somatic copy number alterations (SCNAs), a form of GI, have adverse effects [48]. A comprehensive bioinformatics analysis by Davoli et al. recapitulated compromised cytotoxic activities and CTL infiltration in tumors with high SCNA levels, demonstrating reduced expression of genes encoding components of the T-cell receptor (TCR) complex, as well as genes mediating cytotoxic functions and a proinflammatory TIME [73]. From a therapeutic standpoint, combining the tumor SCNA score with TMB has proven to be a more effective survival predictor for patients receiving immunotherapy than using either biomarker alone. In addition to GI, exogenous carcinogens such as viral infections are implicated in both tumorigenesis and progression by integrating viral genomes into the host [74, 75]. Human endogenous retroviruses are prevalent in malignancies like clear cell renal cell carcinoma (ccRCC) and ovarian cancer, which exhibit upregulated IFN-γ signatures and immune-inflammation [76, 77]. In contrast, non-inflamed tumors are generally characterized by stable genomes and low immunogenicity [13, 78]. The relationship between GI and tumor immunophenotypes is intricate and possibly contingent on a specific tumor type. Gastroesophageal adenocarcinoma (GEA) is a highly heterogenous cancer, and subtypes with MSI or Epstein-Barr Virus positivity demonstrate intense T-cell infiltrates with robust ICB efficacy [79]. However, most chromosomally unstable GEAs are associated with the immune-exclusion phenotype. The diffuse/genome-stable GEA group exhibited enrichment of CD4^+^ T cells rather than cytotoxic CD8^+^ T cells, but the mechanisms are still unknown.

Mutations of the tumor-intrinsic pathways may also indicate a specific immune cell context. For instance, the STING pathway has been linked to the immune-inflamed phenotype as it triggers type I interferons and key chemoattractant for T cell trafficking [80]. A recent report on gastric cancer demonstrated that how human epidermal growth factor receptor-2 (HER2) heterogeneity complicated the TIME is essentially regulated by the STING signaling pathway, whereas HER2-high areas remain immunologically inactivated [81]. Novel cocktail strategies combing the STING agonist bivalent manganese or MSA-2 with anti-TGF-β/PD-L1 bispecific antibody YM101 has successfully inflamed immune-desert tumors, while YM101 alone is insufficient to achieve this outcome [59, 82, 83]. In a T-cell-inflamed mouse model of head and neck squamous cell carcinoma, local injection of a STING agonist into the tumor lesion, followed by anti-PD-L1 treatment, led to successful tumor control and complete rejection [84]. STAT3, an integral signaling node in various oncogenic pathways, is constitutively activated in several malignancies, including lung, breast, and liver cancers. In collaboration with hypoxia-inducible factor-1 (HIF-1), STAT3 orchestrates TWIST1 expression, a crucial marker of epithelial-mesenchymal transition [85]. In immunologically inactive BC, it has been observed that the downregulation of TGF-β in stromal fibroblasts can lead to an upregulation of CXCL1, which then activates STAT3 by acting on CXCR2 in tumor cells [86]. In addition, abnormal activation of WNT/β-catenin pathway has been extensively observed in tumors with high TMB but limited T cell signatures, demonstrating CTL exclusion and blunted immune responses [13, 87–90]. Mechanistically, the overexpression of β-catenin interrupts IFN-γ production in an IL-10-independent manner, as well as reduces the chemoattractant for CD103^+^ dendritic cells (DCs), thereby leading to impaired activation of cytotoxic cells [91]. Moreover, dysregulated WNT/β-catenin signaling is a significant factor in the self-renewal and differentiation of cancer stem cells in various solid tumors [92]. Furthermore, PTEN deletion/PI3K activation have been observed in immune-excluded tumors, leading to resistance towards ICB therapy in both mouse models and human melanoma cases [93]. Promisingly, evidence suggested that a PI3Kβ inhibitor could synergistically enhance tumor control in vivo, holding out hope for leveraging these immune-exclusionary oncogene pathways to bolster immunotherapy efficacy [94].

High levels of GI does not necessarily equate to an immune-inflamed TIME, as TILs are of functional and phenotypic diversity, among which effector CD8^+^ T cells primarily favor the inflamed immunophenotype [95]. Interestingly, Yang et al. have recently identified two patterns of immune “cold”, namely “quantitative cold” and “qualitative cold” in primitive and omental metastatic lesions of ovarian cancer, respectively [96]. The “quantitative cold” TIME is characterized by a high proportion of immunosuppressive regulatory T cells (Tregs) and limited infiltrated CD8^+^ T cells, many of which undergo “exhaustion” due to chronic antigen stimulation within the local ovarian ecosystem [97, 98]. The proportion of Tregs within tumors often exceeds 50% of all T cells, nearly ten times the homeostatic frequency in normal blood and lymphoid tissues [99]. Although Tregs and CTLs can both be recruited to the TIME, Tregs have the potential to impede the further infiltration of their cytotoxic counterparts [100]. Indeed, the ratio of intra-tumoral CTLs to Tregs has been identified as a predictor for immunophenotyping, with higher values often observed in immune-inflamed tumors [101]. The murine model of EMT6 BC is a typical example of immune-excluded phenotype, recent studies indicate that TGF-β supports the dominance of T progenitor-exhausted cells in the intra-tumoral T-cell pool. As the name suggests, the T progenitor-exhausted cells are the originate of exhausted T cells, which are characterized by a progressive loss of effector functions and elevated levels of co-inhibitory receptors, such as PD-1. Previous research by Castiglioni et al. revealed the dual blockade of PD-L1 and TGF-β, along with the ensued IFN-γ signaling activation, yielded an inflamed TIME [26]. In terms of mechanism, the dual targeting strategy facilitated a higher number of functional stem-cell like CD8^+^ T cells to develop along effector differentiation trajectory and the intra-tumoral accumulation of IFNγ^hi^ CD8^+^ T cells triggered TIME-wide IFN licensing, which prompted APM as well as enhanced production of T cell stimulatory cytokines and chemokines [21]. Moreover, CD4^+^ T helper cells can contribute to the reversal of CD8^+^ T cell exhaustion. In an immune-desert murine model of B16-F10 melanoma, Zander et al. revealed that CD4^+^ T cell-derived IL-21 can reprogram CD8^+^ T cells and drive their differentiation into protective cytotoxic CX_3_CR1^+^ CD8^+^ T cells, resulting in a more than two-fold increase in their proportion within the TIME [102]. In contrast, the omental metastatic lesions of ovarian cancer have a preponderance of “bystander” T cells that are only responsive to tumor-irrelevant antigens, which are incapable to initiate tumor-specific immune responses [96]. As indicated by TCR repertoire profiling in ovarian and colorectal cancers, only a minor fraction of CD8^+^ TILs are tumor-specific, while the majority consists of CD39^−^ CD8^+^ “bystander” T cells [97, 103, 104]. Interestingly, “bystander” T cells are exempt from the “exhaustion” program and retain functional memory properties, as they remain ignorant of tumor cells [105]. Both preclinical and clinical investigations have evidenced that “bystander” T cells exhibit low expression level of checkpoint receptors [106]. To leverage the distinctive traits of “bystander” T cells, Chen et al. have developed an engineered oncolytic virus to redirect the antigen specificity of malignant cells to the pre-existing “bystander” T cells, which could synergize with PD-1 and/or PD-L1 ICB therapies for immune-inflamed tumors while sensitize “cold” tumors [107]. These discoveries emphasize that the quality of infiltrates holds equal significance to their quantity, as some TIL-low cancers may have diversified TCR clonality [108, 109]. Functional testing of TCRs has revealed pancreatic ductal adenocarcinoma (PDAC), a typically immune-desert tumor, and high frequencies of tumor-reactive (TR) TCR clonotypes in certain genomically unstable samples. PDAC cases with germline mutations in DNA damage repair genes (such as BRCA1, PALB2) identified TR TCRs, whereas genetically stable samples were mostly dominated by bystander TCR clones [110]. The highest TCR-Vβ diversity, as well as the most skewed TCR-Vβ repertoire (harboring clonally expanded reads) have been observed in the inflamed phenotype, enabling to recognize a wide range of cancerous mutations. Conversely, both of these parameters were generally low in the exclusion and desert phenotypes [58]. Notably, the WNT pathway inversely correlates with the skewing of the TCR repertoire, whereas immune-inflamed tumors are characterized by high TCR clonality independent of GI [22]. Hence, future researchers should adopt a detailed set of criteria that consider the counting, subsets, and functional states of TILs when classifying tumor immunophenotypes. Personalized TR TCR-based adoptive T-cell therapy may offer a perspective for treatment-resistant immune-cold tumors.

Overall, a generalizable link between GI and tumor immunophenotypes remains elusive. A recent large-scale study spanning over thirty-one cancer types revealed that only a quarter of the participants displayed a positive correlation between mutational load and CD8^+^ T cell infiltration, along with optimal ICB responses and prolonged overall survival [111]. In contrast, the remaining large proportion, including prostate and ovarian cancers that are often assumed to be TMB-H, and high-MSI glioblastoma, demonstrate an immune-excluded or -desert phenotype with unfavorable or even negative ICB response rates [38, 112].

### Antigen presenting machinery dictating immune landscape **(**Fig. 3)

**Fig. 3:**
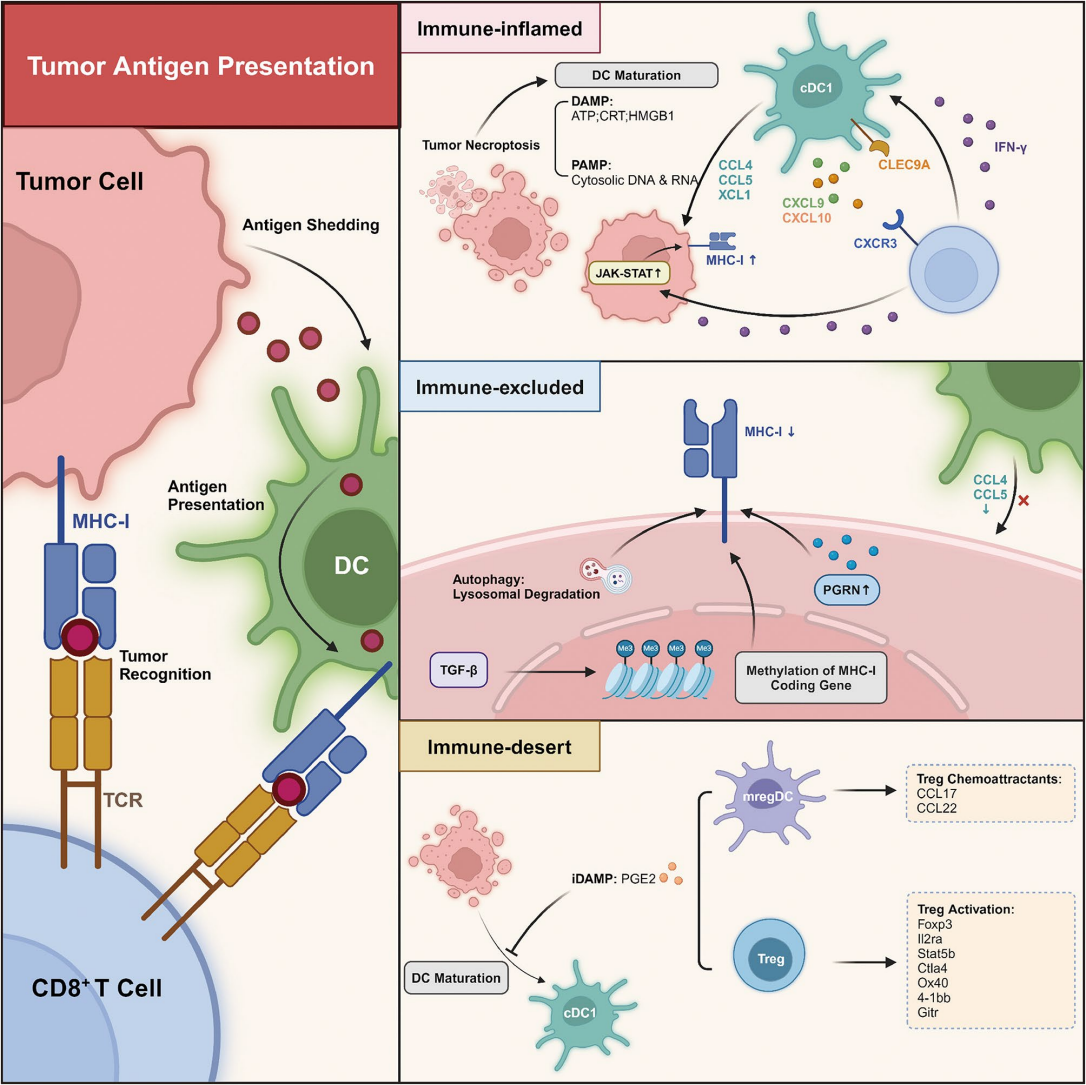
Antigen Presentation Machinery Impact the Landscape of TIME. The antigen presentation machinery involves antigen shedding by tumor cells, this immunogenic signal is then captured and presented by DCs, which subsequently activate CD8^+^ T cells and empower them to recognize and eradicate tumor cells. Elevated immunogenic cell death, specifically necroptosis, is a distinguishing feature of immune-inflamed tumors. Upon detecting DAMPs and PAMPs from dying tumor cells, DCs undergo maturation and are prepared to transmit information for CD8^+^ T cells, in which the immune-enhancing CLEC9A^+^ cDC1s play a major role. As an important mediator, cDC1s migrate to tumor nests through chemoattractant CCL4, CCL5, and XCL1, while simultaneously releasing CXCL9 and CXCL10 to recruit CD8^+^ T cells. In turn, the increased IFN-γ signaling potentiates cDC1s while upregulates tumor MHC-I by triggering the JAK-STAT pathway, thereby forming a positive feedback loop for immune cell infiltration. The immune-excluded tumors generally employ “camouflage strategies” with reduced MHC-I expression. Epigenetically, elevated TGF-β promotes the methylation of MHC-I coding genes. MHC molecules are susceptible to lysosomal degradation via tumor autophagy and the presence of PGRN has been correlated with the downregulation of MHC-I. Moreover, decreased DC chemoattractant also explains the decreased level of T cell infiltration. In the immune-desert TIME, iDAMPs, such as PGE2, can unbalance the antigen presentation machinery by promoting Treg activation, as well as by inducing mregDCs that facilitate Treg infiltration, which collectively lead to a barely inflamed TIME. *Abbreviations*: DC: Dendritic Cell; DAMPs: Pathogen-associated Molecular Patterns; iDAMPs: inhibitory Pathogen-associated Molecular Patterns; PAMPs: Damage-associated Molecular Patterns; CLEC9A: C-Type Lectin Domain Containing 9 A; PGRN: Progranulin; PGE2: Prostaglandin E2

Antigen presenting cells (APCs), indispensable element of the immune system for capturing, processing, and presenting tumor antigens, are rate-limiting for T-cell priming and activation [113, 114]. Among the various APCs, DC are the most potent and are effectively engaged within the inflamed TIME [114]. In murine models, STING agonists have been shown to promote DC maturation and antigen presentation, converting the TIME into an immune-inflamed state [59]. However, a lack of DCs may be the primary reason for the immune-desert phenotype. According to a study on melanoma, the inflamed and non-inflamed subsets exhibited a comparable load of immunogenic antigens, while the latter cohorts were deficient in recruiting and activating Batf3-lineage DCs, the key cell type for the initial cross-priming of anti-tumor CD8^+^ T cells, which implied that any malfunction in the APM could potentially impact the context of the TIME [94].

The APM are activated upon exposure to “danger signals”, including pathogen-associated molecular patterns (PAMPs) and damage-associated molecular patterns (DAMPs) [115]. Typically, DC maturation signals are released by dying tumor cells and act as cues for potential non-self-substances [116]. Subsequently, DCs move towards the draining lymph nodes (dLNs) where they process and load cancer antigens onto MHC-I molecules. This crucial step prepares DCs to present antigens to CD8^+^ T cells [115].

Immune-inflamed tumors generally demonstrate upregulated gene expression of immunogenic cell death, particularly necroptosis. The release of DAMPs from necroptotic tumor cells can potentially amplify IFN-γ production and thus stimulates DC activity, which strongly correlates with the density of intra-tumoral CD8^+^ T cells [117]. C-Type Lectin Domain Containing 9A (CLEC9A), a receptor present on DCs, is required to convey information from necroptotic cells to T cells [118]. In the inflamed TNBC subgroup, a high number of CLEC9A^+^ DCs were found in close proximity to CD8^+^ T cells, indicating the T-cell-activating role of DCs [58]. In contrary, inhibitory damage-associated molecular pattern (iDAMP), such as prostaglandin E2 (PGE2), has been found to dampen the immunogenicity of tumor necroptosis [119]. As observed in murine models of bladder cancer, iDAMP blockade (in the presence of celecoxib or a PGE2 neutralizing antibody) enabled DCs to skew towards immunogenic maturation and successfully transformed the immune-excluded TIME into a T-cell-inflamed pattern [120].

Tumors with immune-inflamed characteristics do not necessarily demonstrate a favorable therapeutic prognosis, as the biological paradox persists: active inflammation often parallels immunosuppression in the TIME [121]. This enigma has been elucidated by several hypotheses involving DCs. Maier et al. elucidated mregDC-Treg axis driven by PGE2-EP2/EP4 signaling. mregDCs, a newly deciphered DC population, are characterized by a homeostatic immunoregulatory gene signature that can restrain immune-enhancing cDC1 and induce anergy in effector T cells [122]. In TIME-inflamed Lewis lung carcinoma-bearing mice, the immune landscape can also be modulated by the regulatory node PGE2-EP2/EP4 signaling, which further potentiates mregDCs and elicits the amplification of CCL22 and CCL17 that expand and activate Tregs [123]. Indeed, maintaining a balanced ratio of cDC1: mregDC is critical for effective cancer elimination, even within an inflamed TIME. Secreted factors such as TGF-β, IL-6 and IL-10 have been shown to hijack this balanced regulatory mechanism, consequently inducing the TIME towards an immunosuppressive profile [124–126].

It has been reported that a subset of DCs migrates to the dLNs and aid in T cell cross-priming, while another group infiltrates the tumor nests to facilitate effector T cell homing and amplify the engraftment of TILs. Chemokines, such as CCL4, CCL5, and XCL1, released from a wide range of cell sources, are key chemoattractants for cDC1s to infiltrate from periphery lymphoid compartments into neoplasms [127, 128]. As observed in treatment-naïve advanced ovarian tumors, CCL5 levels were consistently associated with diffuse T-cell infiltration, which has been proposed to be a targetable factor in transforming immune-desert tumors [129]. Importantly, the dominant chemokines required by DCs to recruit CD8^+^ T cells are those that engage with the chemokine receptor, CXCR3 [130]. In the immune-inflamed TIME, CXCR3-ligands (such as CXCL9 and CXCL10) are predominantly expressed by the CD103^+^ DC population [131]. Aberrant activation of the tumor-intrinsic WNT pathway has been observed to exclude tumor-reactive T cells in melanoma and the immune-excluded subtype of TNBC, resulting in the near-complete absence of T cells within the tumor nest. Mechanistically, the blockade of T cell infiltration could be attributed to the inadequate recruitment of cDC1s and CLEC9A^+^ DCs respectively, which was partly due to the impaired production of CCL4 and CCL5 [94]. Furthermore, β-catenin-expressing tumors also demonstrate failure to facilitate re-expansion of CD8^+^ memory T cells. These findings provide insights into DC-chemoattractant-based reconstitution to address intra-tumoral DC deficiency and restore T cell migration into the tumor parenchyma. Accordingly, a preclinical lung cancer study leveraged nanoparticle-based delivery of CXCL9-11 plasmids towards the TIME, which promoted CD8^+^ T cell infiltration and retarded tumor progression [132]. Meanwhile, studies by Zheng et al. and Terhorst et al. have shown that the upregulation of CCL5 or administration of an XCL1-based vaccibody (bivalent vaccine molecule) improved DC chemotaxis, thereby “heating” a scarcely immunogenic TIME into an “inflamed” TIME by attracting a high number of CD8^+^ T cells [133, 134].

MHC-I molecules are “road marks” for cytotoxic T cells. The functional status of MHC may also contribute to tumor immunophenotypes. As observed in immune-inflamed tumors, elevated IFN-γ upregulates the components of MHC-I by triggering the JAK-STAT signaling pathway [135]. In the immune-excluded subgroup of ovarian cancer, the specific downregulation of MHC-I in the tumor compartment can be partially attributed to epigenetic regulation. Mechanistically, TGF-β promotes DNA methylation, while applying DNA methyltransferase inhibitor has been demonstrated to restore MHC-I in vitro [38]. Furthermore, HLA-I LOH is identified as an independent prognostic marker for patients with the “cold” subtype of TNBC [136]. With impaired DNA double-strand break repair, HLA LOH tumors produce higher levels of neoantigens; however, defective APM demonstrate limited immune selection pressure and an absence of cancer-killing cells within the TIME. As human malignancies progress, tumors may proactively adopt multiple mechanisms to restrict APM and “camouflage” themselves virtually invisible to cytotoxic cells. MHC molecules are susceptible to lysosomal degradation via tumor autophagy. Treatments targeting autophagy can reverse this process with increased CTL infiltration, as shown in a PDAC mouse model [137]. Progranulin (PGRN), a crucial mediator in neurodegenerative diseases, has recently emerged as a TIME modulator. A study of patients with PDAC identified a correlation between PGRN positivity, reduced expression of MHC-I, and deficient infiltration of CD8^+^ T cells. This was further corroborated by PGRN blockade, as it recovered the APM and boosted the cytotoxicity of CD8^+^ T cells [138]. While complete eradication of MHC-I may seem to be a favorable strategy for tumors to maintain a non-inflamed TIME, the immune system has a crucial checkpoint for the loss of MHC-I presentation. Specifically, NK cells act as the “monitor” via “missing self” recognition. In melanoma, the number of NK cells was found to be indicative of both anti-PD-1 efficacy and prognosis, which underscores their potential role against MHC-I-deficient non-inflamed tumors that are refractory to T cell-based regimens [127]. Apart from yielding antigen-loss or MHC-I-negative variants, immune pressure can also select for the outgrowth of tumors cells with activated immune-evasive oncogene pathway such as WNT/β-catenin. Specifically, immune-mediated selection for antigen-loss subclones may occur exclusively in inflamed tumors, which indicates the potential transformation into immune-excluded or immune-desert TIME over time [131].

Overall, any defect in the APM system may lead to a barely inflamed TIME in tumors with high TMB. Indeed, evidence that antigen load may be indistinctive across inflamed-, excluded-, and desert-TIME has holds grounds for exploring novel and general therapeutic strategies to restore DC function and T cell infiltration [94]. Mature DCs are mandatory for an effective anti-tumor response, and focusing on this step in the immune cycle could offer viable treatment options for patients with non-inflamed tumors. DC vaccination therapy yielded encouraging results in a representative immune-desert malignant pleural mesothelioma, as demonstrated in the DENIM trial (NCT03610360) [139]. DCs were ex-vivo cultured and activated before being administered to inflame the TIME, which unleashed the horizon for combination immunotherapy using DC therapy as a backbone.

### T-cell trafficking and infiltration within the TIME **(**Fig. 4)

**Fig. 4:**
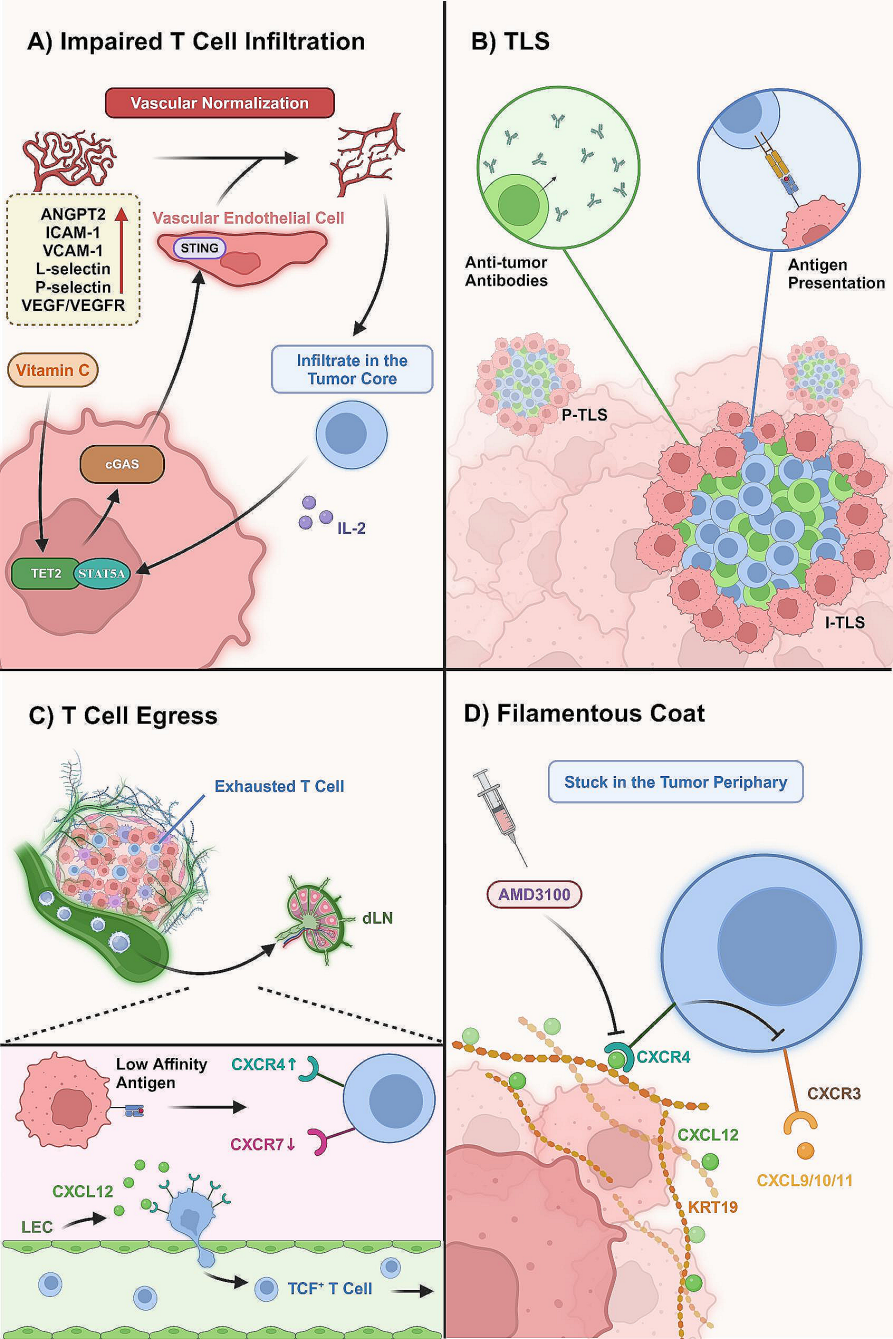
Sustained Intra-tumoral CD8^+^ T cell Infiltration is Integral for the Immune-inflamed TIME. (A) The distorted and leaky tumor vasculatures impede efficient CD8^+^ T cell infiltration. While administration of Vitamin C breaks the vicious circle to normalize vasculatures via the cGAS-STING crosstalk between tumor and vascular endothelial cells. The consequent infiltration of CD8^+^ T cells secrete IL-2 to enhance the crosstalk mechanism, thereby creating a positive feedback loop. (B) TLS, the ectopic lymphoid aggregate, provide alternative infiltration routes for T cells. The specific location and composition of TLSs are determinant for tumor immune phenotypes. I-TLSs are proximate to tumor nests, where anti-tumor antibodies from CD4^+^ T cells and cytotoxicity of CD8^+^ T cells are effectively engaged to eliminate tumor cells, unless TLSs were immature with abundant immunosuppressive Tregs. (C) The T cell egressing mechanism is mediated by lymphatic vasculatures. The upregulated CXCL12-CXCR4 axis between LECs and CD8^+^ T cells expels the TCF1^+^ stem-like population towards dLNs while the exhausted T cell subsets are retained intra-tumorally. This procedure is modulated by tumor-antigen affinity. Specifically, high-affinity antigens downregulate CXCR4 and instead upregulate CXCR7 in CD8^+^ T cells, thereby facilitating T-cell retention. (D) A filamentous network of CXCL12-KRT19 heterodimers coat tumor cells to exclude T cell infiltration. The upregulation of CXCL12-CXCR4 interplay disrupts CXCR3, which is integral for chemoattractant to recruit CD8^+^ T cells. The CXCR4 inhibitor, AMD3100, has been proved to expand intra-tumoral T cell infiltration. *Abbreviations*: cGAS: cyclic GMP-AMP Synthase; STING: Stimulator of Interferon Genes; TLS: Tertiary Lymphoid Structure; I-TLS: Intra-tumoral Tertiary Lymphoid Structure; LEC: Lymphatic Endothelial Cell; TCF1: T Cell Factor 1; dLNs: draining Lymph Nodes

The immunological state of a tumor depends on the extent to which effective tumoricidal cells can access and persist in the tumor parenchyma. The tumor-directed accumulation of immune cells entails sequential interactions that involve tethering, adherence, and migration across specialized post-capillary venules, namely, high endothelial venules (HEV) [140, 141]. To achieve the efficient and durable elimination of malignant cells, cytotoxic T cells must be retained in the tumor parenchyma. The TIME is generally characterized by aberrant destabilized vascularization. Concerted crosstalk between CTLs and tumor vasculature is a determinant for T cell trafficking and infiltration [60].

Emerging evidence highlights the significance of normalized blood vessels in creating an immune-active TIME. Immune-excluded tumors typically contain fewer TILs by sequestering T cells away from their targets. ANGPT2, also known as vascular destabilizing factor angiopoietin-2, was recently observed to destabilize the peripheral vasculature in immune-excluded melanoma, thereby restricting intra-tumoral T-cell accrual [142]. Furthermore, immune-excluded tumors express higher levels of endothelial adhesion molecules (AMs) such as vascular cell adhesion molecule 1 (VCAM-1) at the tumor periphery. The difference in spatial AM expression is postulated to drive T cells to be maintained at the periphery rather than at the tumor core. Interestingly, anti-ANGPT2 treatment improved vascular integrity and decreased AM discrepancy, especially VCAM-1 and L-selectin, which released sequestered T cells from the periphery into the central tumor areas and reversed the immune-excluded phenotype [142]. Targeting the abnormal tumor vasculature system offers promising perspectives for the development of an inflammatory phenotype. A recent murine model-based investigation of liver cancer revealed that Vitamin C is a potential therapeutic agent. Intraperitoneal administration of Vitamin C stimulates teneleven translocation-2 (TET2) and upregulates tumor cyclic GMP-AMP synthase (cGAS). As a result of crosstalk, the STING signaling pathway in endothelial cells is activated, leading to the normalization of tumor vasculature and an increase in transendothelial CTL migration. Consequently, IL-2 produced by infiltrating lymphocytes stimulates tumor STAT5A signaling, which, in turn, synergizes with TET2 to epigenetically elevate tumor cGAS expression, thereby establishing a positive feedback loop [143]. Furthermore, VEGF/VEGFR2 signaling is the most potent driver of dysregulated angiogenesis and has been identified as the key to restore the inflamed TIME [144, 145]. As evidenced by studies of breast and pancreatic cancers, inhibition of VEGF/VEGFR2 signaling improved T cell availability at the tumor core with “vascular normalization” and sprouted HEV [146–149]. In addition, neoropilin-1, a VEGF co-receptor found in tumoral Tregs, has shown double-effect therapeutic benefits. The Treg-specific blockade of neoropilin-1 may attenuate immunosuppressive Tregs and normalize dysregulated angiogenesis, leading to improved CD8^+^ T cell infiltration [150]. Moreover, preclinical investigations have proven that the dual neutralization of ANGPT2 and VEGF altered tumor vasculature effectively and contributes to enhanced anti-tumor immunity [142].

Additionally, the lymphatic system plays an integral role in shaping the TIME, as supported by the positive correlation between lymphatic vessel density and infiltrated cytotoxic T cells in patients with CRC [151]. Steele et al. illustrated that tumor-associated lymphatic vessels can instruct intra-tumoral CD8^+^ T cell repertoire in melanoma [152]. During acute inflammation, CCL21-CCR7 ligation allows for the drainage of naïve T cells back into circulation to maintain immune cell homeostasis [153, 154]. Nevertheless, in tumors, T cells employ a CCR7-independent mechanism to exit through the inflamed lymphatic vasculature, thereby transforming the TIME into an immune-excluded state. Tumor-associated lymphatic endothelial cells upregulate CXCL12 expression, which then interacts with CXCR4 in T cells. Activation of the CXCL12-CXCR4 axis expels TCF1^+^ stem-like T cells, which are integral to sustain the intra-tumoral effector T-cell response, and sequesters them at the tumor periphery, where they are likely to egress [152]. This novel T-cell egressing mechanism is tuned by tumor-antigen affinity. Specifically, high-affinity antigens can downregulate CXCR4 by effector CD8^+^ T cells and instead upregulate the CXCL12 decoy receptor CXCR7 to promote T cell retention. However, this selectively enriched T-cell group was mostly PD-1^+^ TIM3^+^ and LAG3^+^, indicating compromised functionality of retentive T-cells. Similarly, targeting CXCL12-producing fibroblasts unleashed CD8^+^ T cell immunity and persistent tumor control [155]. The interaction between CXCL12 and CXCR4 could otherwise act physically to exclude T cells. As shown in PDAC, CRC, and BC, a filamentous network of CXCL12-KRT19 heterodimers is assembled to coat tumor cells. The CXCL12–KRT19 coating cross-links CXCR4 receptors on adjacent T cells and stimulates a CXCR4 “stop” signal, which plays a dominant role in suppressing T-cell motility and leading to T cell exclusion [156]. As a corollary, targeting the CXCL12-CXCR4 axis could either reduce T cell egression or destroy the filamentous coat that impedes infiltration, thereby expanding the available T cells for immune-noninflamed tumors. Administering AMD3100, a clinically approved CXCR4 inhibitor, has proved increased T cell accumulation within the tumor core [155].

Consistent with these findings, in vivo photoconversion-based analyses of murine models longitudinally examined immune trafficking and egression, which revealed that the majority of CD8^+^ T cells in the tumor deposits undergo an exhausted phenotype within a 72-hour time frame. The intra-tumoral pool of TCF-1^+^ PD-1^+^ cells is continuously replenished by newly entering cells, whereas this population is not retained but amongst the T cells that recirculate to the dLNs, where they can evade chronic antigen exposure to maintain stem-like characteristics [157]. Furthermore, in immune-inflamed TIME, lymphatic vessels can initiate a negative-feedback program upon detecting T cell-derived IFN-γ. Subsequently, elevated lymphatic PD-L1 constraints further CTL accumulation by trapping them in peripheral tissues and creating an excluded infiltrate phenotype [158]. Therefore, it is conceivable that the blockade of PD-L1 could on the one hand rejuvenate the “quantitative-cold” TIME, while simultaneously ensuring sustained infiltration of effector CTLs into the tumor parenchyma.

Tertiary lymphoid structures (TLSs) provide an alternative and efficient route for T cells to migrate towards neighboring tumor nests and proliferate within the TIME, circumventing the conventional vasculature trafficking programs [159]. TLSs are organized ectopic lymphoid aggregates that develop during carcinogenesis and comprise T-cell and follicular B-cell zones [160, 161]. This specialized structure is associated with promising results towards prognosis and ICB therapies, as it provides tumor-responsive T cells with locations for priming, proliferation, and direct migration towards the tumor parenchyma [159, 162]. Indeed, high TLS densities are significantly associated with favorable clinical outcomes across a wide array of solid tumors, including gastrointestinal tumors [163], NSCLC [164], hepatocellular carcinoma (HCC) [165], melanoma [166], BC [167], and CRC [168]. An analysis of NSCLC revealed that patients with stage III disease exhibit fewer TLSs than those with stage II disease, indicating that tumor cells may evade immune responses by disrupting TLS formation during progression [169]. Furthermore, a large-scale retrospective analysis has shown that the number of intra-tumoral TLSs is positively correlated with ICB therapy efficacy, independent of PD-L1 expression [170]. Specifically, for TNBC, a higher abundance of TLS was observed at the periphery of both the inflamed and excluded subtypes, except for the desert immunophenotype [58]. Leiomyosarcoma and intrahepatic cholangiocarcinoma (iCCA), which were previously considered immune-cold and devoid of effector immune cells, exhibited distinctively “hot” subsets characterized by higher frequencies of TLSs [171, 172]. A closer examination suggested that an elevated percentage of stromal cells may impede TLS formation in immune-excluded tumors, consequently reducing the anti-tumor immunity [173]. The function of TLSs may vary across cancer types and depend on spatial arrangements, which classify TLSs into intra-tumoral TLS (I-TLS) and peritumoral TLS (P-TLS). In desmoplastic melanoma, I-TLSs are in closer proximity to tumor cell clusters, contributing to an immunologically inflamed TIME, unlike in non-desmoplastic melanoma, where TLSs are embedded peritumorally [174]. In contrast, in CRC, a high density of P-TLS confers an immune-active TIME and improved prognostic value, whereas I-TLSs are perfused by immunosuppressive Tregs [173]. Ding et al. revealed that the maturity of TLS is reliant on CD4^+^ Bcl6^+^ follicular helper T cells [172]. It is speculated that in the absence of this cell group, P-TLS may remain immature and dysfunctional, potentially destroying the formation of an inflamed TIME by expanding Tregs in I-TLSs. In addition to T cells, the role of TLS-resident B cells in orchestrating the immune-inflamed TIME has recently emerged as a focal point of research [175–177]. Interestingly, Vanhersecke et al. proposed that the presence of mature TLS correlated with favorable outcomes in tumors featuring high CD8^+^ T cell infiltration, whereas patients with limited CD8^+^ T cell infiltration failed poorly, irrespective of TLS status [170]. This observation indicates that CD8^+^ T cells are essential but not adequate for initiating a persistent anti-tumor immune response, necessitating close cooperations with B cells. Researchers have uncovered high expression of both MHC class I and class II molecules in B cells within TLSs, highlighting their proficiency in antigen presentation [166]. Moreover, TLS can facilitate the differentiation and maturation of B cells into plasma cells, which can produce tumor-specific antibodies that attach to tumor cells [178]. Investigations into PDAC have elucidated that this process can be explained by the synergistic interactions involving TGF-β signaling, the expression of the B cell chemoattractant CXCL13 by tumor-reactive T cells, and the supportive role of fibroblast-derived TGF-β, which collectively enhance the activation of B cells [179]. Further analysis of ccRCC suggested that these mature plasma cells within the TLS are guided by CXCL12^+^ fibroblasts to migrate deeper into tumor foci while producing IgG and IgA antibodies and boosting anti-tumor effects [180]. The subsequent formation of antigen-antibody complexes could be internalized by DCs and favor efficient antigen presentation to T cells, which allows enhanced activation of CD8^+^ T cells, particularly in the context of ICB therapies [181–183]. Collectively, these findings add an important facet to leverage the TLS as a biomarker for identifying potential patients who may benefit from ICB therapies. Currently, clinical trials in patients with sarcoma are exploring this innovative approach (NCT02406781; NCT04095208) [170]. According to a study by Hammerl et al., however, the presence or density of TLSs in TNBC are independent of survival rates, even when stratified by immunophenotypes, which warrants pertinent research to determine their contribution in shaping the TIME [58].

To date, it is unclear whether the egress mechanism of lymphatic vasculature is a bone or bane because it not only transports functional T cells away from tumors but is also likely to reinvigorate or recirculate the suppressed, exhausted, or naïve T cells, though to a relatively lesser extent. Furthermore, infiltrating T cells migrate to distant metastatic tumors and dLNs, underscoring a mechanism by which tumor-experienced effector T cells boost tumoricidal efficacy at secondary tumor sites [184]. Techniques such as photoactivation strategies and advanced imaging are emerging and are supportive of unraveling the advantageous or detrimental effects of T cell egress from intra-tumoral regions [185].

### Immune-suppressive cells extinguishing immune inflammation

The cancer ecosystem recruits and domesticates an array of immunoregulatory cells to mold tumor immune phenotypes by interacting with various components within the TIME. For example, melanoma and PD-L1^+^ myeloid cells, particularly macrophages and DCs, collaboratively form thin and continuous sheaths along the invasive border, thereby excluding CTLs [186]. In general, the major immunoregulatory cell populations, including myeloid-derived suppressor cells (MDSCs), neutrophils, tumor-associated macrophages (TAMs) and Tregs, play a significant role in reshaping the TIME either by quantitative superiority over cancer-killing cells or by modulating CTLs through diverse mechanisms [187].

MDSCs are a heterogeneous population of immature myeloid cells at various differentiation stages and can be categorized into monocytic MDSCs (M-MDSCs) and polymorphonuclear MDSCs (PMN-MDSCs), according to their phenotypic and morphological similarities to monocytes and granulocytes, respectively [188, 189]. As the key mediator orchestrating immunosuppression across solid tumors, the intra-tumoral enrichment of MDSCs has been linked to an unfavorable prognosis [190]. Indeed, the progenitors of MDSCs activate multiple signaling pathways that promote their amplification and inhibit further differentiation, with the majority converging on the JAK-STAT pathway [191], which upregulates immunosuppressive mediators such as reactive oxygen species (ROS), arginase, and inducible nitric oxide synthase (iNOS) [192]. Tumor-mediated PMN-MDSCs primarily inhibit effector T cells via ROS, whereas M-MDSCs mainly suppress T cells via arginase and iNOS [189, 193]. MDSCs are negatively associated with CTLs, where decreased MDSCs result in an increased intra-tumoral frequency and amplified tumoricidal effect on CD8^+^ T cells [194, 195]. An investigation of PDAC has unveiled that CXCL1 is a molecular “switch” between the inflamed and the non-inflamed TIME. As a tumor cell-intrinsic regulator, non-inflamed tumor-derived CXCL1 signaled to increase the infiltration of MDSCs and simultaneously expels DCs and T cells, driving resistance to immunotherapy [196]. CCL2 triggers the release of tumor-toxic granules in CD8^+^ T cells and NK cells, whereas MDSC-produced reactive nitrogen species can nitrify CCL2 (N-CCL2) [197]. Studies on human colon and prostate cancers demonstrated that N-CCL2 entraps T cells in the stroma, creating an immune-excluded TIME [198]. However, the disruption of CCL2 nitration promotes favorable lymphocyte infiltration in preclinical models. Additionally, plasma membrane expression of the metalloprotease ADAM17 by M-MDSCs has been proposed to downregulate L-selectin in T cells, thereby impairing their extravasation from the lymphatic system and migration into tumor lesions [199]. This scenario is further supported by a study using a B16 mouse model, which showed that L-selectin deficiency resulted in a limited number of tumor-infiltrating T cells [200]. Moreover, compared to circulating MDSCs, tumor-residing MDSCs demonstrate higher PD-L1 expression in various murine models and cancer patients, which interacts with T cells to induce their anergy [201–203]. Therefore, strategies to counteract the tumor-supportive activities of MDSCs may offer significant therapeutic benefits. In a preclinical model of immune-desert bladder cancer, the concomitant application of MDSC blockade with radiotherapy synergistically enhanced tumor-infiltrating CTLs, underscoring a breakthrough in MDSC-targeted therapy for remodeling the tumor immunophenotype [204].

Tumor cells may exploit neutrophil biology in their own interest, including hijacking the formation of neutrophil extracellular traps (NETs). NETs are web-like chromatin structures composed primarily of DNA extruded from the nuclei of neutrophils [205]. Research has indicated that the NET-DNA structure serves as a protective shield against cytotoxicity for tumor cells by physically blocking direct interactions with tumoricidal cells [206]. Confocal imaging of tumors has revealed that NETs form a barrier at the tumor-stroma interface. In recent studies, researchers have discovered that a direct chemotactic stimulant, the tumor-produced cytokine Chitinase-3-like 1 (Chi3l1, YKL-40/CHI3L1 in humans), promotes NET formation in TNBC [207]. Chi3l1 co-opts neutrophils to mediate stromal restriction of T cells, whereas ablation of Chi3l1 ameliorates tumor growth and enhances the ICB response. In the context of pancreatic cancer, γδ T cell-secreted IL-17 contributed to NET formation, which further suppressed CD8^+^ T cell recruitment and conferred the immune-excluded phenotype of PDAC [208], while IL-17 neutralization has allowed spatial redistribution of CTLs that favored proximal migration to tumor nests. Furthermore, the presence of PD-L1 in NETs has a broader impact by shifting T cells into the exhausted phenotype, inducing a “quantitative cold” state [209]. As shown in invasive bladder cancer, the administration of DNase I to digest NETs also inflamed the TIME with effective CD8^+^ T cells accumulation [210].

TAMs are also important players in the TIME, representing the predominant population of tumor-infiltrating immune cells (over 50%) in various cancer types, such as melanoma, renal cancer, and CRC [211]. TAMs are highly plastic cells that exhibit functional heterogeneity in response to various stimuli [212]. Typically, TAMs can be categorized into two classes: M1 and M2 [213]. Classically activated M1 macrophages are primarily involved in proinflammatory responses. Similar to DCs, M1 TAMs are capable of phagocytosing tumor-associated antigens, albeit to an inferior extent [214], and can serve as APC to induce specific anti-tumor immune responses [213]. Conversely, M2 TAMs undergo alternate activation and are primarily involved in anti-inflammatory responses. Both M1 and M2 TAMs are present throughout all stages of tumor development, with M1 TAMs dominating in the early stages and M2 TAMs prevailing as the tumor advances [215]. M1 TAMs progressively shift towards M2-polarized TAMs with tumor progression, whereas an elevated frequency of M2 TAMs correlates with an unfavorable prognosis. Intravital imaging studies of the TIME indicated that antigen-specific CD8^+^ T cells tended to localize in TAM-rich areas and positive correlations existed between the infiltration and exhaustion of CD8^+^ T cells with TAMs [216, 217]. Research of melanoma models indicated that the highest level of exhaustion in cytotoxic T cells is present near the macrophage barrier, where macrophages are plentiful and have increased opportunity to interact with T cells within the “effective interaction distance”, typically defined within a radius of less than 20 μm [218]. TAMs and CD8^+^ T cells are shown to engage in a weakly stimulatory, yet persistent antigen-specific synaptic contacts that initiate T cell exhaustion program and may lead to a “quantitative cold” state [214]. Thereafter, exhausted T cells concurrently form a self-enforcing feedback loop, which is exacerbated under hypoxic conditions, particularly at the tumor core, to expand the intra-tumoral pool of TAMs via secreting CSF1, CCL3-5. Moreover, TAMs, by CCL18, can function to recruit naïve CD4^+^ T cells with the potential to differentiate into Tregs via secreting TGF-β and IL-10 [219, 220]. As reported in studies on ovarian cancer patients and murine gastric cancers, TAMs can attract mature Tregs into the TIME through the production of chemokines CCL20 and CCL22, which cooperate to suppress CD8^+^ TILs [221, 222]. Furthermore, a recent investigation on CRC has revealed that the TIME specifically endowed TAMs with elevated inhibitor of differentiation 1 expression, which interacted with STAT1 to suppress the chemoattractant CCL4, thereby excluding CD8^+^ T cells from tumor parenchyma [223]. Macrophage-coated tumor clusters (MCTC), distinctive spatial structures characterized by abundant macrophages surrounding tumor clusters, have been identified in HCC. MCTC is a prevalent structure that was also observed in 35.5% of BC samples and 23.5% of lung squamous carcinoma samples, which impedes CD8^+^ T cell infiltration and relegates them to the tumor periphery [224]. In terms of the mechanism, the tumor-derived macrophage-associated lectin Mac-2 binding protein could orchestrate the recruitment of the M2 macrophage subpopulation and enhance cellular adhesion, leading to the formation of this robust immunosuppressive barricade. Therefore, converting M2 TAMs into anti-tumor M1 subtypes is a promising immunotherapeutic approach for the treatment of solid tumors [225]. The application of chimeric antigen receptor macrophages (CAR-Ms) in preclinical studies has demonstrated M1 TAM polarization, improved phagocytosis of tumor cells, and restored tumoricidal function of CD8^+^ T cells [226, 227]. Compared with CAR-T therapies, CAR-M has particular advantages in terms of its capacity to migrate and penetrate the immunosuppressive TIME of solid tumors [228]. However, this innovative technology remains immature in the clinical setting. In contrast to the preclinical results where CAR-Ms reprogrammed the TIME, the TIME was capable of steering tumor-resident CAR-Ms towards a tumor-supportive phenotype, highlighting the need for further exploration into the underlying mechanism [229].

Immune-suppressive cells are intricately linked. Research of melanoma-bearing mice has shown that indoleamine 2,3-dioxygenase (IDO) can orchestrate both local and systemic immunosuppression through the expansion, infiltration, and function of MDSCs within the TIME, in a manner dependent on Treg recruitment [230, 231]. Interestingly, inhibiting IDO or depleting Tregs decreased intra-tumoral MDSCs and reversed immune suppression. Studies have revealed a correlation between MDCSs and Tregs in multiple cancers such as metastatic prostate cancer, glioblastoma, and renal cell carcinoma [232]. Moreover, IDO-deficient mice demonstrate retardation of lung tumor progression, with MDSCs exhibiting impaired immunosuppressive ability due to IL-6 attenuation [233]. Previous research has also proposed that IDO can induce and activate Tregs; however, the mechanisms underlying Treg-mediated MDSC recruitment and activation are not completely understood [234]. In addition, Treg-DC interactions have been shown to disrupt CTL-DC engagement, leading to unfavorable enrichment of inactivated T cells. Treg’s contact-dependent ligation with DCs also generates metabolic destructions that contribute to a “quantitative cold” TIME, the consequent IDO production by DCs catabolizes the essential amino acid into suppressive metabolites including kynurenine, which in turn activates Tregs and MDSCs [235]. Administering the IDO1 inhibitor, LW106, to melanoma cells resulted in a reduction in tumor-associated stromal cells and collagen deposition, consequently eliciting CTL infiltration [236]. These findings provide a compelling rationale for using IDO inhibitors as adjuvants to convert immune-cold tumors by highlighting the significant association between immunosuppressive cells and IDO in the TIME.

### Barriers blocking T-cell infiltration

Currently, the exploration into how physical traits of cancer impact the immune landscape is still in its infancy. Solid stress, a compressive mechanical force mediated by the proliferation of cancer cells and desmoplasia of the ECM, has been shown to deter lymphocyte ingress and facilitate immune exclusion [237]. As measured in metastatic lymph node lesions, solid stress surges towards the lesion center. This modification of the TIME impairs lymphocytic trafficking by reducing the number of HEVs, particularly those expressing peripheral node addressin, the lymphocyte-homing receptor on endothelial cells. Immune exclusion is primarily observed in tumors with a collagen-rich ECM that features compact and linearly aligned fibers in the tumor stroma [238]. As shown in PDAC, stromal components account for > 90% of the tumor mass [239]. Analysis of human using real-time imaging of T-cell dynamics has revealed dense fibers oriented parallel to the interface between the tumor and stroma [34]. This distorted stromal architecture creates a rigid barrier that peritumorally compartmentalizes the cancer-attacking cells. It also obstructs the diffusion of cytokines and chemokines, which are crucial to recruit and activate T cells. Moreover, T cells may adhere to dense collagen, which could spark rapid motility along the collagen highway, rendering them distracted from the durable and serial killing of their targets [240]. Additionally, collagen can function as an immunosuppressive ligand for leukocyte-associated immunoglobulin-like receptor-1, curtailing cytotoxicity and inducing T-cell exhaustion [241]. Eliminating discoidin domain receptor 1, the collagen receptor responsible for collagen fiber realignment, promotes CTL infiltration in murine models of immune-excluded tumors such as metastatic urothelial cancer and TNBC [33, 242]. In melanoma, the administration of recombinant hyaluronan and proteoglycan link protein 1 led to a highly “basket-weaved” ECM structural pattern, which closely resembled that of normal epithelial tissues and was associated with increased T cell infiltration [241, 243]. The abundance of metalloproteinases, which are critical enzymes involved to degrading collagen and restructuring ECM fibers, was found to have an elevated infiltration advantage [244]. Alternatively, metalloproteinase-cleaved collagen fragments also activate integrin-dependent T cell motility, indicating that collagen elements can create chemotactic gradients that guide CTLs towards intra-tumoral areas [245]. However, it is yet to be determined whether collagen-degraded fragments can mobilize T cells at a high velocity and disrupt their engagement with cancer cells, or whether they can guide them to tumor cell-dense areas. Overall, these findings merit further attention regarding the critical role of ECM modulation processes in immune cell distribution, and whether targeting them could guarantee more frequent interactions with tumor cells remains unknown.

Cancer-associated fibroblasts (CAFs) play a prominent role in ECM deposition and remodeling that is endemic to immune-excluded tumors [246]. Transformed from quiescent normal fibroblasts, this immune population exhibits distinguished propagation and migration abilities. In fact, CAFs strongly demonstrate inter- and intra-tumor heterogeneity. Fibroblasts expressing fibroblast activation protein (FAP) are responsible for producing and organizing fibrous materials (such as fibronectin and collagen), thereby driving T cell marginalization and restricting their contact with cancer cells [247]. It has been noted that ECM-associated functions are predominantly executed by FAP^+^ CAFs, which are identified in regions where the matrix is densely deposited [34]. A recent study of NSCLC discovered multiple layers of FAP^+^ CAFs surrounding the tumor border, driving T-cell marginalization through the deposition and alignment of type XI and XII collagens. Another distinctive CAF subset with positive expression of myosin heavy chain 11 lines around a single layer and was strongly associated with immune exclusion [248]. These CAF subpopulations are characterized by high levels of periostin (POSTN) as well. Pan-cancer analysis of TCGA database has provided evidence that individuals with high POSTN or FAP expression are associated with a high Tumor Immune Dysfunction and Exclusion (TIDE) score, indicating greater potential for immune evasion and unfavorable prognosis from immunotherapy [249]. In murine BC models, nitric oxide underlies the stromal effects of CAFs featuring FAP and podoplanin (PDPN) positivity. Mechanistically, nitric oxide generated by FAP^+^ PDPN^+^ CAFs initiates TCR nitration in neighboring T-cells, which consequently leads to desensitization [250]. Similarly, N-CCL2 is implicated in the peritumoral entrapment of CTLs in the stroma. However, CAF heterogeneity may explain the contradictory outcomes of previous studies on stromal depletion. In PDAC, the composition of the immune cell infiltrates is dictated by two distinct CAF subtypes: POSTN^+^ CAFs and PDPN^+^ CAFs [251]. Tumors lacking PDPN^+^ CAFs feature an immune-cold phenotype, and tumors with an adequate presence of PDPN^+^ CAFs are associated with T cell infiltrates, unless abundant with POSTN^+^ CAFs, which preferentially favor macrophage chemotaxis by activating the Akt signaling but exclude T cells from infiltration [249]. Consistently, the positive correlation of PDPN^+^ CAFs and intra-tumoral T cells has been observed in TNBC, in which the immune-inflamed subtype is predominant [252]. Nevertheless, genetic ablation of CAF-derived POSTN has demonstrated accelerated tumor growth because it is essential for the formation of tumor capsules, indicating that certain POSTN^+^ CAFs may be protective against tumor progression [253].

Senescence is a hallmark of cancer [254]. In addition to the heterogeneity of CAFs, the contribution of senescence programs should be carefully determined. Evidence suggests that senescent CAFs, which can be derived from various CAF subpopulations, reshape the TIME via an intricate tumor-CAF interplay. Essentially, the evolution of the senescent TIME is inextricably correlated with a shift in fibroblast behavior [255]. Senescent cancer cells have limited or null proliferative capacity; however, they emit a collection of pleiotropic cytokines, chemokines, growth factors, and matrix-modifying factors, which are referred to as the senescence associated secretory phenotype (SASP), to directly initiate stromal cell senescence [256, 257]. In the context of tumor progression, CAFs experience long-term induction of the SASP, which leads to immunosuppressive cell infiltration, including MDSCs, Tregs, and M2 macrophages [258, 259]. In addition, a study on ccRCC showed that immune exclusion-related signaling activity is upregulated along with the activation of the senescent program in CAFs, resulting in adverse prognostic implications [260].

Of note, TGF-β is a pivotal upstream mediator in CAF-associated exclusionary stromal reaction. Immune-excluded tumors including CRCs and urothelial cancers have demonstrated increased levels of TGF-β-driven CAF gene expression program [33, 261]. In a model of pancreatic cancer with selective Treg deletion, the absence of Treg-derived TGF-β1 hinted towards decreased collagen synthesis by CAFs, subsequently leading to a greater T cell influx [262]. Beyond Tregs, TGF-β can be produced by various cell subsets within the TIME, including tumor itself and CAFs [261]. In addition, cancer-derived exosomes also carry nucleic acids or proteins such as surface-bound TGF-β1 to promote CAF generation [261]. Congruently, in the typical hypoxic TIME, increased TGF-β stimulates CXCL12/CXCR4 signaling via HIF-1α in both cancer cells and CAFs [263], and the elevation of CXCL12/CXCR4 axis serves the downstream role to recruit and activate CAFs, thereby driving matrix production and subsequent stromal T-lymphocyte exclusion [264]. The administration of AMD3100, a clinically approved CXCR4 inhibitor, has been shown to decrease fibrosis and alleviate solid stress that physically repels T cells in the stroma [265]. Furthermore, the ROS-producing enzyme NADPH-oxidase-4 (NOX4) is also recognized to act downstream of TGF-β1 and modulates CAF differentiation in multiple cancers. Investigations applying GKT137831 (Setanaxib), a small molecule inhibitor of NOX4/1, suggested that targeting NOX4 can revert CAFs to the “normalized” phenotype and increase intra-tumoral CD8^+^ T cell density as a result [266]. In tumor models featured with TGF-β activity in CAFs, using pan-TGF-β antibody has successfully enabled T cell infiltration [33]. A study interrogating TGF-β neutralization has revealed significant reduction of ECM density accompanied with a shift of tumor fibroblast landscape, which involved an expansion of IFN-licensed CAFs. This distinctive CAF population is marked by strong responses to IFN signaling, either by demonstrating increased MHC molecules and enhanced APM system, or by promoting T-cell infiltration via CXCR3 [267]. Despite “heating” the TIME, the strategy of targeting TGF-β seems unable to reverse the established CAF phenotype [266]. Indeed, the effective inhibition of TGF-β1 downstream mediators, such as the CXCL12/CXCR4 axis and NOX4, holds great potential for overcoming T-cell exclusion while maintaining a remarkable safety profile. TAMs are another promoter of fibrillar collagen by stimulating CAFs [268]. The coexistence of tumor-specific SPP1^+^ TAMs and CAFs has been identified at the tumor boundary in the TIME of CRC and HCC. Blocking or specifically deleting SPP1 in the macrophages of murine models disrupts the desmoplastic microenvironment, leading to reduced CAF infiltration and enhanced cytotoxic T-cell infiltration [269, 270]. A study of PDAC revealed a feedback mechanism between macrophages and fibroblasts. Fibroblast-secreted IL-33 stimulates macrophages to produce CXCL3, which may act as an IL-33 imitator that targeted CXCR2 in stromal fibroblasts, thereby promoting CAF transition and collagen III generation [271].

Evidence is prominent for trials combining therapeutic approaches to address both the tumor stroma and malignant cells, which may unleash further advantages **(**Table 2**)**. Recently, prior administration of FAP-CAR T cells has shown improved efficacy for subsequent tumor antigen (mesothelin)-targeted CAR T cells or anti-PD-1 antibody therapy [247]. On the one hand, the removal of FAP^+^ CAFs disrupts the dense matrix and stromal border around tumor clusters, thereby facilitating the trafficking of cytotoxic effector cells and their direct communication with cancer cells. On the other hand, FAP-CAR T cells encourage T cell infiltration by inhibiting the CXCL12/CXCR4 axis and reducing chemokines (such as CCL3/4/5) which suppress the recruitment of immunosuppressive myeloid cells within the TIME. However, FAP is expressed in certain healthy tissues; thus, complete ablation of FAP is impractical and can potentially cause toxicity such as cachexia and anemia [272]. As a study on BC suggested, different FAP^+^ stromal cells may exhibit dissimilar functions, phenotypes, and distributions; therefore, FAP-based treatments require careful assessments [273]. To circumvent these puzzles, “reprogramming” CAFs may be a prospective alternative. One possible solution is to administer Vitamin D agonists to restore quiescent normal fibroblasts, which is currently under investigation in an ongoing clinical trial (NCT03520790) [274]. Furthermore, blocking CTLA-4 on CD8^+^ T cells counteracted CAF-mediated T-cell exclusion without affecting CAF levels [33]. The build-up of CAF-induced solid stress highlights formidable challenges for CTL infiltration and immunotherapies that depend on either endogenous or adoptively transferred T cells. Losartan has previously been demonstrated to alleviate solid stress by reducing collagen levels while increasing normalized HEVs, resulting in effective T-cell entry [247].

**Table 2:** Clinical trials targeting CAFs in different tumor indications

Categorization	Tumor Indications	Clinicaltrials.gov Identifier	Treatment Arms	Current Status
CAF Normalization	Metastatic Pancreatic Cancer	NCT03520790	Paricalcitol (Vitamin D receptor agonist) Gemcitabine Nab-paclitaxel	Phase 1/2
	Pancreatic Cancer	NCT03331562	Paricalcitol (Vitamin D receptor agonist) Pembrolizumab	Phase 2
	Squamous Cell Carcinoma of Head and Neck	NCT05323656	Setanaxib (GKT137831, NOX4 inhibitor) Pembrolizumab	Phase 2
Targeting Downstream Effectors	Metastatic Pancreatic Cancer	NCT02734160	Galunisertib (LY2157299, TGF-β Receptor I Kinase Inhibitor) Durvalumab	Phase 1
	Urothelial Carcer	NCT04064190	Vactosertib (TGF-β Inhibitor) Durvalumab	Phase 2
	Breast Cancer; Lung Cancer; Hepatocellular Cancer; Colorectal Cancer; Pancreatic Cancer; Renal Cancer	NCT02947165	NIS793 (anti-TGF-β monoclonal antibody) Spartalizumab	Phase 1
	Thymic Cancer; Thymoma	NCT04417660	Bintrafusp Alfa (M7824, bifunctional fusion protein targeting PD-L1 and TGF-β)	Phase 2
	Urothelial Cancer	NCT04501094	Bintrafusp alfa (M7824, bifunctional fusion protein targeting PD-L1 and TGF-β)	Phase 2
	NSCLC	NCT03631706	Bintrafusp alfa (M7824, bifunctional fusion protein targeting PD-L1 and TGF-β)	Phase 3
	Solid Tumors	NCT04291079	SRK-181 (anti-latent TGFβ1 monoclonal antibody) Anti-PD-(L)1 antibody therapy	Phase 1
	NSCLC	NCT04515979	Vactosertib (TEW-7197, TGFβ1 inhibitor) Pembrolizumab	Phase 2
	Pancreatic Cancer	NCT02907099	BL-8040 (CXCR4 antagonist) Pembrolizumab	Phase 2
	Metastatic Pancreatic Adenocarcinoma	NCT02826486	BL-8040 (CXCR4 antagonist) Pembrolizumab Chemotherapy of Onivyde	Phase 2
	Pancreatic Adenocarcinoma; Metastatic Ovarian Serous Adenocarcinoma; Colorectal Cancer Metastatic	NCT02179970	Plerixafor (CXCR4 antagonist)	Phase 1
	Solid Tumors	NCT02754141	BMS-986,179 (antibody inhibiting CD73 enzymatic activity) Nivolumab rHuPH20	Phase 1/2
	Renal Cell Carcer	NCT05501054	Ciforadenant (adenosine A2a receptor antagonist) Ipilimumab Nivolumab	Phase 1/2
	Renal Cell Cancer; Metastatic Castration Resistant Prostate Cancer	NCT02655822	Ciforadenant (adenosine A2a receptor antagonist) Atezolizumab	Phase 1
Targeting CAF-derived ECM Proteins	NSCLC	NCT02346370	PEGylated Recombinant Human Hyaluronidase (PEGPH20, to degrade major ECM component hyaluronan) Docetaxel	Phase 1
	Pancreatic Cancer	NCT03634332	PEGylated Recombinant Human Hyaluronidase (PEGPH20, to degrade major ECM component hyaluronan) Pembrolizumab	Phase 2
	Lymphoma; Melanoma; Renal Cell Carcinoma	NCT00001683	COL-3 (NSC-683551, matrix metalloproteinase inhibitor)	Phase 1
Depleting CAF	Breast Cancer; Cancer of Head and Neck	NCT02627274	FAP-IL2r (RO6874281, immunocytokine consisting of interleukin 2 variant targeting FAP) Trastuzumab Cetuximab	Phase 1
	Metastatic Melanoma	NCT03875079	FAP-IL2r (RO6874281, immunocytokine consisting of interleukin 2 variant targeting FAP) Pembrolizumab	Phase 1
	Renal Cell Carcinoma	NCT03063762	FAP-IL2r (RO6874281, immunocytokine consisting of interleukin 2 variant targeting FAP) Atezolizumab Bevacizumab	Phase 1
	Metastatic Colorectal Cancer	NCT04826003	FAP-4-1BBL (RO7122290, FAP targeted 4-1BB Ligand) Cibisatamab Obinutuzumab	Phase 1/2
Inhibiting CAF Activation	Ovarian Cancer	NCT01778803	Defactinib (VS-6063, Focal Adhesion Kinase Inhibitor) Paclitaxel	Phase 1
	Pancreatic Cancer	NCT02546531	Defactinib (VS-6063, Focal Adhesion Kinase Inhibitor) Pembrolizumab Gemcitabine	Phase 1

Overall, rather than intrinsic disorders of CD8^+^ T cells, the transformed stromal microarchitecture that favors peritumoral retention of immune cells may play a more critical role for immune-excluded tumors. Further research is required to elucidate how specific therapeutic approaches manipulate ECM distribution and T-cell infiltration. Analyzing stromal heterogeneity across cancer types and treatments from a high-dimensional perspective is essential to comprehending the diverse roles of cell components within the stroma and identifying novel treatment strategies.

### Metabolic disorders shaping the TIME

Transformation of the metabolic landscape in the TIME has been deemed as an established hallmark of cancer [275]. Malignant cells and tumor-residing cells dynamically engage in spatial and temporal cooperations. The interconnected network for resource acquisition and exchange in TIME necessitates a holistic outlook. Tumor cells proactively exploit and manipulate local metabolite availability, establishing their dominance for energy and nutrients over non-cancerous cells [276]. For instance, under the hostile hypoxic condition of CRC, the upregulation of stanninocalcin 2, a glycoprotein hormone involved in glutamine or glucose deprivation, has been implicated in the preparation of tumor cells adapted to metabolic shifts, thereby promoting tumor progression [277]. TILs are subjected to the incurred metabolic stress, which drives the derangements of their metabolic programs. In CRC, with the combined application of multiplexed ion beam imaging by time of flight (MIBI-TOF) and antibody-based single-cell metabolic regulatory profiling (scMEP), Hartmann et al. uncovered a CD8^+^ T cell subset with CD39 and PD-1 expression that was metabolically repressed and excluded from the tumor-immune boundary, indicating niche-driven modulation of immune cell distribution and functionality [278]. Indeed, high levels of hypoxia, lactate, acidification, as well as deficiency of essential amino acids, all modify the immunometabolism of immune cells and have been appreciated as determinants of a diminished intra-tumoral T cell pool [279].

#### Hypoxia driving multifaceted TIME perturbations

Solid stress and hypoxic state of the TIME are interdependent. Specifically, insufficient oxygen is a common characteristic across a spectrum of solid tumors, primarily because of limited oxygen delivery caused by aberrant neovascularization and robust matrix desmoplasia, both of which are indicative of high mechanical compression. Proliferative malignant cells outstrip blood supply and compete with neighboring immune cells for oxygen and nutrients [280]. It should be noted that the oxygen distribution within tumors is spatially heterogeneous and influenced by their proximity to blood vessels [281]. Recent studies on immunometabolism have suggested that oxygen tension is a tightly linked parameter of the tumor immune landscape. The hypoxic TIME undergoes drastic metabolic perturbations, leading to a metabolic barrier to efficient tumor elimination. According to Sugiura et al., immune-desert tumors employ metabolic adaptations against efficient T cell functionality and proliferation [282]. As a proof-of-concept, a preclinical investigation into prostate cancer revealed that hypoxic niches displayed resistance to CTL infiltration, even in the presence of CTLA-4 and PD-1 dual-blockade, unless a hypoxia-reliving therapeutic approach was applied [283]. Intriguingly, supplemental oxygen can markedly alleviate the hypoxic conditions of TILs and promote T cell infiltration in murine models [284].

Importantly, multifaceted hypoxic signatures are associated with the immune-cold subtypes of various tumors [285, 286]. Specifically, glioblastoma is notorious for its immunologically “desert” TIME, where hypoxic niches, located distal to the incompetent vasculature, are found to entrap TAMs and CTLs and subsequently reprogram them into immunosuppressive state [287]. Mechanistically, TAM-derived CCL8 and IL-1β are essential hypoxic-niche factors to further attract and retain more cancer-killing cells, creating a vicious circle of this distinct temporospatial pattern. As hypoxia level rises, both in vivo and in vitro studies have reported impaired IFN-γ-dependent MHC-I expression, which is reversible once the oxygen-level is restored [288, 289]. Additionally, the hypoxia-induced ecto-nucleotidase CD39 coordinates with CD73 on Tregs to digest ATP and ADP into adenosine [290], which suppresses CTLs and induces a T cell exhaustion program [291]. The hypoxic TIME may also disadvantage T cells into terminal-exhausted state by driving mitochondrial stress and further facilitates the “quantitative cold” immunophenotype. ZipSeq, a spatial transcriptomic technique, maps exhaustion-related gene expression patterns and shows enrichment in hypoxic areas within the TIME [292].

Moreover, hypoxia is responsible for aggravating ECM remodeling by inducing enzymes, such as lysyl oxidases and collagen prolyl 4-hydroxylase, ultimately excluding T cells [293]. Previous studies have also demonstrated that long-term hypoxia in tissues can enhance TGF-β signaling. Indeed, the downstream HIFs and TGF-β are reciprocally induced, contributing to the disorganized ECM structure that further exacerbates solid stress and hypoxia [38, 294]. As revealed in colorectal adenocarcinoma, a string of signaling activation is involved in hypoxia-induced biological functions, including WNT, HIF-1, and ECM-related pathways. These signaling pathways collectively remodel the TIME in colorectal adenocarcinoma, which tends to present an excluded immunophenotype [295].

#### Aerobic glycolysis inducing lactate accumulation and acidosis in the TIME **(**Fig. 5)

**Fig. 5:**
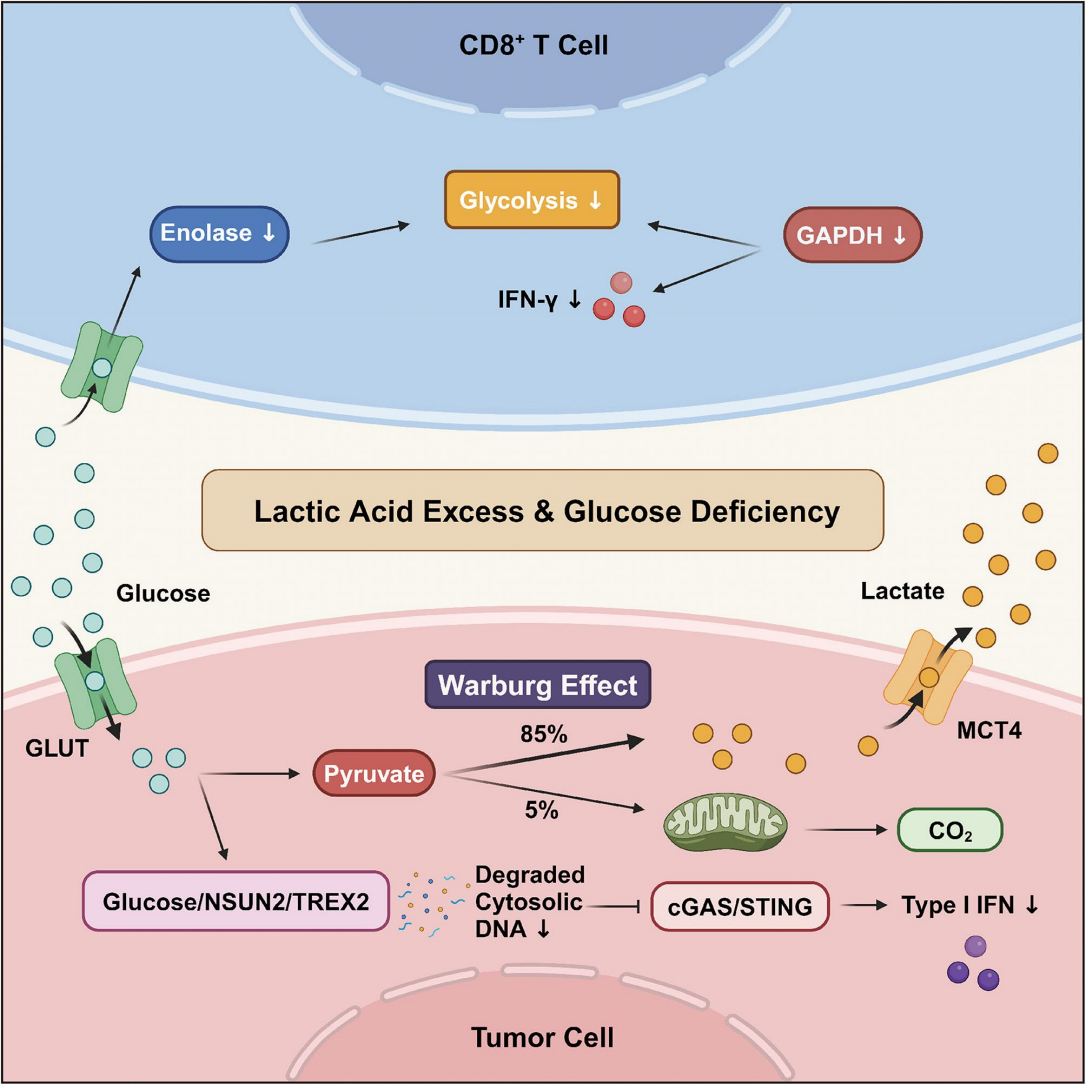
Exacerbated Glucose Competition between Tumor and CD8^+^ T cells in the Hypoxic TIME. Immune-noninflamed (immune-excluded and -desert phenotype) tumors undergo metabolic adaptations to compete for more oxygen and glucose against CD8^+^ T cells. Mechanistically, immune-noninflamed tumors enhance glucose uptake by upregulating the glucose transporter GLUT. In the hypoxic TIME, this metabolic competition is further intensified by the Warburg effect, as tumor cells prioritize aerobic glycolysis, converting glucose into a substantial amount of lactate. Accordingly, the lactate transporter MCT4 is upregulated in the hypoxic condition, which facilitates the efflux of lactate and subsequently promotes TIME acidification. Glucose also serves as signaling molecule, as the glucose/NSUN2/TREX2 axis can shut off the cGAS/STING pathway, which is crucial for T cell recognition and infiltration in the immune-inflamed TIME. Conversely, CD8^+^ T cells in immune-noninflamed tumors demonstrate constrained glucose metabolism characterized by insufficient uptake and impaired activity of key enzymes involved in the glycolytic pathway, such as enolase and GAPDH. Furthermore, the reduced activity of GAPDH can compromise the generation of proinflammatory cytokine IFN-γ at the posttranscriptional level. *Abbreviations*: GLUT: Glucose Transporter Type 1; MCT4: Monocarboxylate Transporter 4; NSUN2: NOP2/Sun RNA Methyltransferase 2; TREX2: Three Prime Repair Exonuclease 2; GAPDH: Glyceraldehyde-3-phosphate Dehydrogenase

The hypoxic TIME demonstrates a shift towards glycolytic metabolism owing to the potent activation of HIF-related genes. Metabolic reprogramming promotes aerobic glycolysis, also termed as “Warburg effect”, which is a well-recognized hallmark of cancer [296, 297]. Regardless of the oxygen level, tumor cells prioritize glycolysis as an energy resource and readily convert glucose into large amount of lactate [298]. In this context, HIF-1α induces the overexpression of monocarboxylate transporter 4 (MCT4) on tumor cells, which facilitates the draining of lactate into the TIME so as to maintain intracellular PH homeostasis. In lung adenocarcinoma, the serine/ threonine kinase STK11 (also called LKB1) is a frequently mutated tumor suppressor gene that has been identified as the main driver of the inert immune-cold phenotype, despite the presence of a paradoxically high TMB due to LKB1 deficiency [299]. LKB1-mutant lung adenocarcinoma presents drastic metabolic alterations with elevated MCT4 expression, enhanced MCT4-dependent lactate secretion polarizes macrophages into the immunosuppressive M2 subtype and hampers T cell function as a consequence [300, 301]. In this sense, targeting the MCT4 lactate transporter offers a therapeutic route for overcoming the “cold” immune phenotype with restored CTL frequency and activity.

Immunometabolism studies have revealed that anti-tumor immune cells share comparable nutrients as cancerous cells, giving rise to a competitive dynamic between them. Similarly, T cells display glycolysis characteristics that sustain proliferation and increase fitness under extremely oxygen-deficient conditions [296]. Because of the metabolic tug-of-war during rapid cancer progression, T cells are unable to eliminate their targets with full potential. Notably, the level of glucose transporter type 1 (GLUT1) in tumors is inversely associated with CD8^+^ T cell infiltration and survival in squamous cell carcinoma [302]. However, T cells in certain cancers demonstrate an intrinsic impairment in glycolysis. In human ccRCC, T cells may downregulate GAPDH, which leads to insufficient glucose uptake and use, even under the nutrient-replete conditions [303]. In this case, IFN-γ production can be compromised given GAPDH is exactly engaged in the posttranscriptional regulatory process of this proinflammatory cytokine [304]. Moreover, glucose deprivation of T cells may also induce the anergic exhausted state, leading to the “quantitative cold” TIME with impaired functional properties [305]. Neutralizing tumor acidity has been shown to improve response to ICB therapies [306]. Nevertheless, immunosuppressive Tregs can be invigorated in this therapeutic context due to lactate-induced PD-1 expression on Tregs, potentially compromising the efficacy of immunotherapies [307]. Glucose deprivation may not be a universal characteristic for malignant tumors. In a study of melanoma, neither a deficiency in GLUT1 expression nor an inability of CTLs to uptake glucose from the TIME was observed. Rather, CD8^+^ T cells demonstrate constrained glucose metabolism owing to the impaired activity of enolase, a critical enzyme in the glycolytic pathway. ICB therapies retard melanoma progression by increasing CTL infiltration with restored enolase activity, which was observed either in the recently activated CTLs or in the newly tumor-infiltrating T cells instead of reactivating enolase in the pre-existing CTLs [308, 309]. Moreover, in experimental settings, an adequate amount of ^18^F-fluorodeoxyglucose is generally provided, which does not accurately reflect glucose availability within the TIME or the proficiency of glucose uptake by cells.

Admittedly, most investigations deem aberrant glucose metabolism as the key mediator of the TIME immune status. However, a recent study has suggested that glucose can directly disengage the immune response as a signaling molecule [310]. The glucose/NSUN2/TREX2 axis restricts cytosolic dsDNA accumulation, which shuts off cGAS/STING signaling for apoptosis, and consequently thwarts both CD8^+^ T cell recognition and infiltration. Targeting this axis offers promising insights to resetting immune-cold tumors with aberrant glucose signaling into the inflamed tumors. This novel paradigm can precondition refractory immune-cold tumors such as prostate cancer and luminal subtype BC for enhanced efficacy in subsequent immunotherapies.

#### Amino acids deprivation (Fig. 6)

**Fig. 6:**
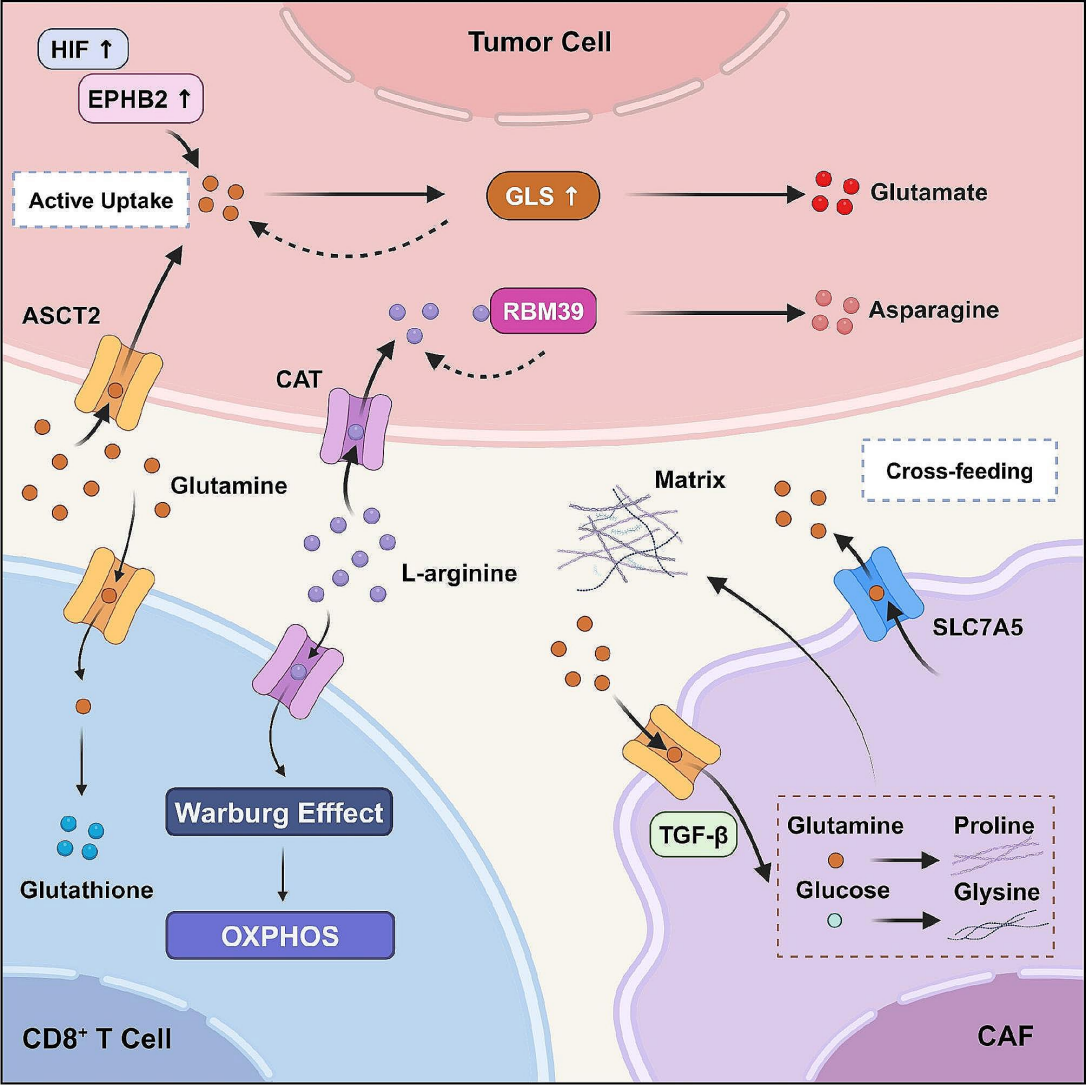
Tumor Cells Outcompete CD8^+^ T Cells in Essential Amino Acid Consumption to Promote Immune-excluded or -desert TIME. In immune-noninflamed (immune-excluded and -desert phenotype) TIME, tumors acquire large amount of glutamine either by overexpressing glutamine transporters or by cross-feeding from other cells such as CAFs. The former mechanism involves upregulated GLS in glutaminolysis, which in turn accelerates glutamine uptake by tumors. In the hypoxic TIME, HIF can equip tumor cells with upregulated EPHB2 and activate GLS promoter to accumulate glutamine. Moreover, CAFs can secrete glutamine via SLC7A5, which can be utilized by tumor cells to their advantage, especially for immune-excluded ones. Comparatively, CD8^+^ T cells demonstrate limited access for glutamine in immune-noninflamed tumors, the consequent deficiency of antioxidant glutathione has been associated with reduced T-cell density and impaired function. L-arginine is critical for CD8^+^ T cells to shift from the Warburg effect to OXPHOS, however, the lion’s share of extracellular L-arginine is taken up by immune-noninflamed tumors through CAT. Then, L-arginine binds with RBM39 for further asparagine synthesis, which in turn enhances arginine uptake. Furthermore, CAFs, the predominant component of the immune-excluded TIME, are significantly involved in depriving CD8^+^ T cells of essential amino acids as well. Mechanistically, CAFs favor migrating towards glutamine-high areas and subsequently provide guidance for tumor cells. Meanwhile, the upregulated TGF-β signaling coordinates CAFs to produce matrix proteins that are enriched with proline and glycine, which are transformed from glutamine and glucose respectively, thereby aggravating immune-exclusion as a result. *Abbreviations*: CAFs: Cancer-associated Fibroblasts; GLS: Glutaminase; HIF: Hypoxia-inducible Factor; EPHB2: EPH Receptor B2; SLC7A5: Solute Carrier Family 7 Member 5; OXPHOS: Oxidative Phosphorylation; CAT: Cationic Amino Acid Transporter; RBM39: RNA-binding Motif Protein 39

Compared to glucose that has garnered significant attention, researchers have only recently begun to delve into the impact of amino acids on tumor immune compartments. In a study on colon cancer, Rathmell et al. examined the metabolic features of various cell components in the TIME and discovered that CD8^+^ T cells were not deficient in glucose, in contrast, cancer cells outcompeted in glutamine consumption four-fold higher than that of CD8^+^ T cells [311]. In vivo metabolic tracer experiments revealed that TIME-residing cells do not consume nutrients proportionately. Specifically, cancer cells take up the lion’s share of glutamine, whereas cells of the myeloid lineage, such as macrophages, are the primary consumers of glucose [311]. Furthermore, cancer cells may benefit from the cross-feeding of amino acids by other tumor-residing cells [312]. In ovarian cancer, CAF-secreted glutamine via solute carrier family 7 member 5 can be leveraged by cancer cells to fuel progression [313]. Therefore, one can envision that the unbalanced amino acids partitioning between cancer and immune cells would favor malignant cells while shaping the different immunophenotypes.

As with glucose, there may be competition for glutamine between cancer and immune cells, creating a scenario in which tumor cells outperform the uptake of local glutamine. T cells rely heavily on extracellular glutamine availability rather than de novo synthesis upon activation [314]. Accumulating evidence has indicated that glutamine plays immunomodulatory role for TILs, as a higher rate of glutamine use correlates with a lower apoptosis rate of themselves [286]. Investigations on human basal-like BC have proposed an inverse relationship between tumor glutamine metabolism and T cell cytotoxicity markers [315]. Furthermore, glutamine starvation hampers nucleotide synthesis and cytokine production, thereby impairing T cell activation and proliferation [314]. Genetic ablation of glutaminase (GLS), a critical enzyme that boosts conversion into glutamate, in tumor cells could lead to increased glutamine concentration and improved T-cell infiltration within tumor nests. Therefore, glutamine use by tumor cells is a potential immunoregulatory metabolic checkpoint that alters the characteristics of TILs. Under hypoxic conditions, HIF can equip cancer cells with the upregulated EPH receptor B2 for the uptake and accumulation of glutamine [286]. Additionally, the major glutamine transporter, ASC amino-acid transporter 2 (ASCT2), is overexpressed in cancers, such as melanoma and prostate cancer [316]. For TNBC that is specifically “glutamine-addicted”, tumor cells exhibit elevated levels of ASCT2 as well as GLS [317]. Likewise, studies have found in hypoxic CRC, HIF-1 activates the promoter of GLS-1, thereby accelerating the rate-limiting step in glutaminolysis [318]. In addition to cancer cells, the anabolic program within CAFs can also deprive TILs of glucose and glutamine. Especially for immune-excluded tumors, TGF-β coordinates CAFs towards robust production of matrix proteins, which are highly enriched with proline and glycine [319]. To this end, CAFs increase the consumption of these raw materials, facilitating the synthesis of glycine from glucose via the serine biosynthetic pathway, and generating proline from glutamine. Indeed, evidence suggests that CAFs are sensitive to glutamine concentration within the TIME, favoring their transfer towards glutamine-high regions [320]. The glutamine distribution gradient increases towards the peripheral area in BC, which may explain why CAFs tend to retain in the peripheral stroma and induce immune-exclusion [320]. In turn, CAFs direct tumor cells towards these glutamine-enriched territories, contributing to tumor progression and metastasis [294]. Additionally, elevated tumor niche stiffness was found to mechanoactivate glycolysis and glutamine metabolism in cancer cells and CAFs via the YAP/TAZ pathway, creating a vicious circle of tumoricidal cell immunometabolism [321]. Activated T cells increase glutamine intake and metabolism to support various biological processes, including mitochondrial anaplerosis, nucleotide synthesis, amino acid generation, and redox balance, without which T cell-mediated immune responses are impaired [322].

For TNBC that are characterized by glutamine-addicted metabolism, targeting cancer metabolic reprogramming to reverse the tumor “glutamine steal” phenomenon, while sparing anti-tumor T cells, may hold therapeutic insights. In a TNBC murine model with specific GLS loss on tumor cells, studies revealed an elevated glutamine concentration in the tumor stroma that promoted the synthesis of glutathione, a major cellular antioxidant, in T cells to improve intra-tumoral CD8^+^ T cell density and functionality. Moreover, in poorly immunogenic and “non-inflamed” EGFR-driven lung cancer, oral administration of a specific glutamine antagonist against cancer cells, JHU083, facilitated the enrichment of CD8^+^ T cells [323, 324]. JHU083 is capable of converting immunosuppressive MDSCs and TAMs into tumor-destroying proinflammatory phenotypes [325]. Furthermore, pharmacological blockade of ASCT2 with V-9302 preferentially inhibits glutamine use in cancer cells, which upregulates the immune checkpoint factor PD-L1 by impairing the activity of Sarco/ER Ca^2+^-ATPase (SERCA). Therefore, co-treatment with glutamine depletion and anti-PD-L1 antibody, therefore, is feasible and represents a promising strategy with synergistic anti-tumor effects and increased T- cell infiltration [326]. Taken together, an integrated understanding of glutamine metabolism in the TIME is of utmost importance as it provides crucial pathways that could be targeted by novel strategies. Indeed, it has the potential to yield a two-pronged attack that bolsters tumoricidal immune responses, while concurrently crippling tumor metabolism.

In addition to glutamine, L-arginine is known to regulate glycolysis and mitochondrial activity by interacting with transcriptional regulators that are essential for the function of TILs. Studies have indicated that intracellular L-arginine can prompt a metabolic transition from glycolysis to oxidative phosphorylation (OXPHOS) in activated T cells, thereby counteracting the Warburg effect and decreasing lactate production in the TIME [327]. One underlying mechanism is that elevated L-arginine may upregulate the serine biosynthesis pathway, which can facilitate OXPHOS [327]. Tumors actively take up arginine via cationic amino acid transporters, as documented in the context of hepatocellular carcinoma, which bind to the RNA-binding motif protein 39 (RBM39) for further metabolic reprogramming. Importantly, RBM39-mediated elevation of asparagine synthesis promotes arginine uptake, thereby forming a positive feedback loop to sustain arginine accumulation and oncogenic metabolism [328]. Additionally, arginase 1-expressing myeloid cells, which are prevalent in immune-cold tumors, primarily deplete T cells of arginine, resulting in the blockade of TCR expression and anti-tumor response [329]. PDAC is characterized by abundant infiltration of myeloid cells, leading to uncontrolled metabolism of L-arginine by arginase 1 and iNOS activity. Consequently, the generation of reactive nitrogen species establishes a chemical barrier that shields tumor cells from CTL recognition and entrance into the tumor core [330]. Because locally restoring the intra-tumoral L-arginine concentration can be challenging, Canale et al. devised an innovative engineered bacterium that can colonize tumor nests and convert the metabolic waste product ammonia to L-arginine, resulting in an increased frequency of TILs and additive effects with ICB treatments [331].

#### Metabolism-targeted interventions

Targeting dysregulated metabolic alterations in the TIME serves as an intriguing avenue for eliminating tumors. Therapeutic approaches involve targeting cancer cell metabolism to transform the TIME into a more conducive one for T cell efficacy. The GLS inhibitor CB-830 is currently undergoing phase 1 clinical trials (NCT02071862, NCT03875313, and NCT02861300) with inspiring outcomes. Glycolysis inhibitors, such as 2-Deoxyglucose, have garnered increasing popularity for the treatment of CRC at the preclinical stage [332]. Because TNBC also displays high glycolytic rates, the GLUT1 inhibitor BAY-876 has shown significant effectiveness in counteracting tumor proliferation. However, targeting glycolysis is not universally effective, as glycolysis serves as a crucial source of energy over various cell populations, and tumor cells also exhibit heterogeneity in carbohydrate metabolism. For instance, the infusion of 2-Deoxyglucose demonstrated an undesirable hypothalamus response in certain clinical trials due to its non-specificity [333]. To address this issue appropriately, a future strategy would be to introduce an agent that enhances the metabolic competitiveness of tumoricidal T cell populations within the TIME. Nutrient supplementation was also instrumental. Remarkably, a two-fold increase in intracellular L-arginine levels via oral administration has been demonstrated to promote the generation of central memory-like T cells in a murine model, which, when combined with adoptive T-cell therapy and immunotherapy, could potentially inflame the TIME and revive anti-tumor activity [327, 334]. A recent study outlined an interrelationship in which glutamine primes the tumor immunity of cDC1 [335]. Adequate intra-tumoral injection of glutamine licenses cDC1 for efficient antigen presentation, which has implications for augmenting CD8^+^ T cell activation and overcoming ICB resistance.

However, randomized clinical trials on metabolism-targeted interventions have yielded unsatisfactory outcomes across the board [336]. Despite limited success in tumor elimination, these treatments may facilitate tumor progression by driving metabolic bypass, adaptation, differentiation, and therapeutic resistance. Li et al. discovered that, in response to glutamine starvation, the stress-induced transcription factor DNA damage induced transcript 3 is activated to promote glycolysis, thereby generating ATP to sustain tumor growth during metabolic stress [337]. In addition to this complexity, metabolism-targeted drugs may also have “off-target” effects on non-cancerous cells. As such, these findings underscore the necessity of delineating the biological similarities and variations between target cancer cells and the other tumor-residing counterparts.

## Conclusions

The TIME is a complex ecosystem composed of various cell types whose functionality and spatiality are typically hijacked to create a tumor-supportive and immune suppressive environment. ICB therapies have shown promise for patients with immune-inflamed tumors; however, such success is yet to be achieved for most immune-excluded or-desert tumors [338]. Exploring treatment strategies that can inflame the TIME serves as putative option for curing patients with cancer. The non-comparative phase II TONIC trial (NCT02499367) revealed that metastasized TNBC can be converted into an immune-inflamed state when preconditioned with chemotherapeutic agents, indicating the plasticity of immunophenotypes and the potential of priming non-inflamed tumors to favor immunotherapy [27]. Moreover, the majority of research conducted thus far has predominantly leveraged the pre-treatment state of the TIME to predict ICB responses, oversimplifying the fact that the TIME dynamically evolves alongside the tumor. Mariniello et al. demonstrated the superiority of sequential administration, in which chemotherapy is followed by PD-1 blockade, over a concomitant strategy [339]. This finding provides a strong rationale for delving deeper into the seemingly nuanced alterations in the TIME during treatments. To date, since holistic knowledge of the local TIME and overall tumor ecosystem is lacking, experiments and clinical trials on tumor immunophenotypes still yield certain contradictory outcomes.

The concept of personalized cancer immunotherapies has been ambitiously advocated. Precise immunophenotype-based stratification is integral to determine tailored therapeutic approaches in clinic settings, as highlighted by the IMvigor210 trial (NCT02951767 and NCT02108652), wherein a reduced panfibroblast TGF-response gene signature was linked with atezolizumab efficacy, but only restricted to immune-excluded tumors [33]. As prominent challenges persist in the accuracy and availability of TIME decoding technologies, their practical implementation has not been scaled up. Of note, our team comprehensively reviewed emergent multiomics technologies for deciphering the TIME in the context of TNBC [340]. Apart from conventional methods to evaluate the TIME, such as IHC and flow cytometry, revolutionary techniques are now available that approach towards a 3D-dimensioned standpoint and even integrate temporal analysis of the dynamic TIME [341]. Spatial transcriptomics combines high-resolution spatial architectures with single-cell RNA sequencing or single-nucleus RNA sequencing data, providing researchers with high throughput information across various biological samples [342]. Based on next-generation sequencing, spatially resolved transcriptomics technology has emerged in a timely manner with a unique position to delve into specific spatial structures in the TIME [160]. The integration of artificial intelligence has recently ushered in a new epoch to elucidate TIME patterns and establish feasible predictive models with improved objectivity, consistency, and comprehensiveness in clinical and investigational contexts [343–345]. Machine learning has facilitated the discovery of in-depth biomarkers and intercellular relationships within the TIME. For instance, skin cutaneous melanoma with an increased Banfield Raftery index (TIL cluster count) hinted towards favorable survival, whereas BC with a high Ball Hall index (TIL cluster extent) correlated with inferior prognosis [346, 347]. In laboratories, because cell line models cannot accurately recapitulate the crisscross communication network within the TIME, the advent of tumor organoids or patient-derived xenografts may fully capture the tumor ecosystem and better inform clinical trials [348]. Overall, by determining the spatiotemporal dynamics of the TIME, we can explore deeper into the contributors of different immunophenotypes and guide personalized precision medicine in the future.

## Data Availability

No datasets were generated or analysed during the current study.

## Abbreviations

ICB: Immune checkpoint blockade
ACT: Adoptive cell therapy
CAR: Chimeric antigen receptor
PD-1: Programmed cell death 1
PD-L1: Programmed cell death ligand 1
CTLA-4: Cytotoxic T lymphocyte-associated antigen-4
TIME: Tumor immune microenvironment
ECM: Extracellular matrix
TILs: Tumor-infiltrating lymphocytes
CRC: Colorectal cancer
MSI: Microsatellite instability
sTILs: Stromal tumor-infiltrating lymphocytes
APM: Antigen presentation machinery
IFN: Interferon
IHC: Immunohistochemistry
3D: Three-dimensional
NSCLC: Non-small cell lung cancer
BC: Breast cancer
TNBC: Triple negative breast cancer
LS: Lynch syndrome
GI: Genomic instability
TMB: Tumor mutational burden
TMB-H: High tumor mutational burden
SCNA: Somatic copy number alteration
TCR: T-cell receptor
ccRCC: Clear cell renal cell carcinoma
GEA: Gastroesophageal adenocarcinoma
HER2: Human epidermal growth factor receptor-2
HIF-1: Hypoxia-inducible factor-1
DC: Dendritic cell
Tregs: Regulatory T cells
PDAC: Pancreatic ductal adenocarcinoma
TR: Tumor-reactive
APC: Antigen presenting cell
PAMP: Pathogen-associated molecular pattern
DAMP: Damage-associated molecular pattern
dLN: Draining lymph node
CLEC9A: C-type lectin domain containing 9 A
iDAMP: Inhibitory damage-associated molecular pattern
PGE2: Prostaglandin E2
PGRN: Progranulin
HEV: High endothelial venules
ANGPT2: Angiopoietin-2
AM: Adhesion molecule
VCAM-1: Vascular cell adhesion molecule 1
TET2: Teneleven translocation-2
cGAS: Cyclic GMP-AMP synthase
TLS: Tertiary lymphoid structure
HCC: Hepatocellular carcinoma
iCCA: Intrahepatic cholangiocarcinoma
I-TLS: Intra-tumoral tertiary lymphoid structure
P-TLS: Peritumoral tertiary lymphoid structure
MDSC: Myeloid-derived suppressor cell
TAM: Tumor-associated macrophage
M-MDSC: Monocytic myeloid-derived suppressor cell
PMN-MDSC: Polymorphonuclear myeloid-derived suppressor cell
ROS: Reactive oxygen species
iNOS: Inducible nitric oxide synthase
N-CCL2: Nitrify CCL2
NET: Neutrophil extracellular trap
Chi3l1: Chitinase-3-like 1
MCTC: Macrophage-coated tumor cluster
CAR-M: Chimeric antigen receptor macrophage
IDO: Indoleamine 2, 3-dioxygenase
CAF: Cancer-associated fibroblast
FAP: Fibroblast activation protein
POSTN: Periostin
TIDE: Tumor immune dysfunction and exclusion
PDPN: Podoplanin
SASP: Senescence associated secretory phenotype
NOX4: NADPH-oxidase-4
MIBI-TOF: Multiplexed ion beam imaging by time of flight
scMEP: Single-cell metabolic regulome profiling
MCT4: Monocarboxylate transporter 4
GLUT1: Glucose transporter type 1
GLS: Glutaminase
ASCT2: ASC amino-acid transporter 2
SERCA: Sarco/ER Ca2^+^ -ATPase
OXPHOS: Oxidative phosphorylation
RBM39: RNA-binding motif protein 39
